# Progression from oligoarticular to polyarticular psoriatic arthritis and apremilast as a disease modifier: Novel insights from FOREMOST

**DOI:** 10.1093/rheumatology/keag351

**Published:** 2026-07-01

**Authors:** Laura C Coates, Dafna D Gladman, Joseph F Merola, Ulrich Mrowietz, April Armstrong, Xenofon Baraliakos, William Tillett, Mitsumasa Kishimoto, Jyotsna Reddy, Lichen Teng, Hamid Amouzadeh, Cynthia Deignan, Philip J Mease, Laure Gossec

**Affiliations:** Nuffield Department of Orthopaedics, Rheumatology and Musculoskeletal Sciences, University of Oxford, Oxford, UK; Schroeder Arthritis Institute, Krembil Research Institute, Toronto Western Hospital, Toronto, Ontario, Canada; Department of Medicine, Rheumatology, University of Toronto, Toronto, Ontario, Canada; Department of Dermatology and Department of Medicine, Division of Rheumatology, UT Southwestern Medical Center, Dallas, TX, USA; Psoriasis Center, Department of Dermatology, University Medical Center Schleswig-Holstein, Kiel, Germany; Dermatology, University of California Los Angeles, Los Angeles, CA, USA; Rheumazentrum Ruhrgebiet, Herne, Germany; Ruhr-University, Bochum, Germany; Department of Life Sciences, University of Bath, Bath, UK; Department of Nephrology and Rheumatology, Kyorin University School of Medicine, Tokyo, Japan; Amgen Inc, Thousand Oaks, CA, USA; Amgen Inc, Thousand Oaks, CA, USA; Amgen Inc, Thousand Oaks, CA, USA; Amgen Inc, Thousand Oaks, CA, USA; Swedish Medical Center/Providence St. Joseph Health and University of Washington School of Medicine, Seattle, WA, USA; Sorbonne Université, INSERM, Institut Pierre Louis d’Epidémiologie et de Santé Publique, Paris, France; Rheumatology Department, AP-HP, Pitié Salpêtrière Hospital, Paris, France

**Keywords:** apremilast, psoriatic arthritis, PsA, oligoarticular, polyarticular, disease activity, disease progression

## Abstract

**Objectives:**

Data regarding progression from oligoarticular to polyarticular psoriatic arthritis (PsA) are sparse. Using FOREMOST, we identify progression predictors and evaluate apremilast’s treatment effect in early PsA.

**Methods:**

FOREMOST (NCT03747939) randomized N = 308 patients with early (mean duration, 9.9 months) PsA and limited joint involvement (>1 to ≤4 swollen and >1 to ≤4 tender joints; not confirmed by imaging) to apremilast or placebo for 24 weeks, followed by open-label apremilast through week 48. Multivariable logistic regression modelled predictors of progression from oligoarticular (≤4 active [swollen and/or tender] joints) to polyarticular (>4 active joints) PsA at week 16 and the effect of apremilast. Disease progression, disease activity, clinical signs/symptoms and tolerability were summarized through week 48 for *N* = 291 patients receiving ≥1 apremilast dose (from randomization or switched from placebo).

**Results:**

Most (268/308 [87.0%]) patients had oligoarticular PsA at baseline; 25.1% (59/235) progressed to polyarticular PsA by week 16 (data as observed). Apremilast reduced odds of progression *vs* placebo by 58% (odds ratio [OR; 95% CI]: 0.42 [0.22, 0.77]). In placebo-treated patients, being female, being csDMARD-naïve, and having dactylitis significantly increased odds of progression (OR: 3.35 [1.20, 9.34], 3.42 [1.18, 9.93], and 9.26 [1.32, 65.09], respectively). Apremilast treatment for up to 48 weeks maintained low rates of disease progression and improvements in disease activity/clinical signs and symptoms, with no new safety signals.

**Conclusion:**

Initiating apremilast treatment during the early, oligoarticular phase of PsA reduced disease activity and delayed progression to polyarticular disease, with benefits maintained with up to 48 weeks of treatment.

**Trial registration:**

ClinicalTrials.gov; NCT03747939.

## Graphical abstract

**Graphical Abstract:**
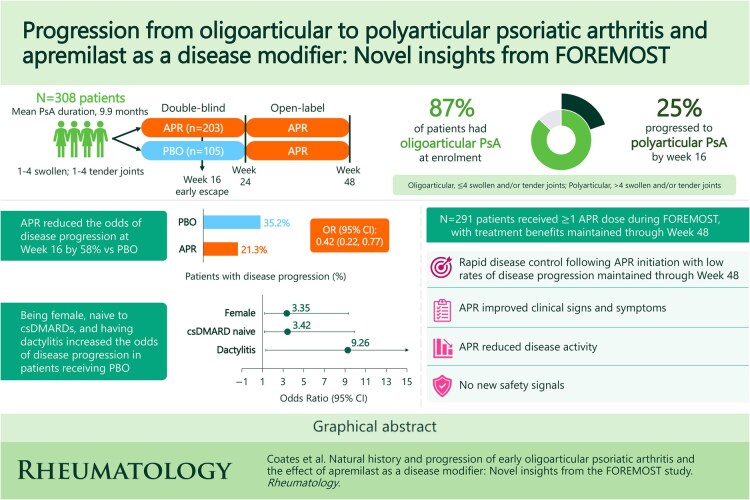

Rheumatology key messages

- In FOREMOST, both large and small joints contributed to early oligoarticular PsA.
- Being female, being csDMARD-naïve, and having dactylitis, predicted progression from oligoarticular to polyarticular PsA.
- Apremilast halved the odds of progression, with treatment benefits maintained up to 48 weeks.

## Introduction

Psoriatic arthritis (PsA) is typically classified as oligoarticular (≤4 active joints) or polyarticular (>4 active joints) [1–3]. While oligoarticular PsA is common in early disease, occurring in 40–60% of patients with PsA [2–7], little is known about this phenotype, including the most commonly involved joints. Moreover, data regarding evolution to polyarticular disease is sparse, with varied findings [2, 8]. In a Canadian cohort of early (<12 months) PsA, 39% (75/192) of patients with oligoarticular PsA experienced polyarticular progression at 9–10 years follow-up [2]. In a United Kingdom (UK) cohort (average disease duration, 12 years), 75% (18/24) of patients with oligoarticular PsA experienced polyarticular progression [8].

Timely diagnosis and therapeutic intervention at the oligoarticular stage of PsA could attenuate, delay or prevent progression to polyarticular disease, which is associated with joint damage [9–11]. However, complexity and heterogeneity of PsA make diagnosis challenging, causing delay in initiating effective treatments [12]. In addition, with clinical trials focusing on established polyarticular PsA, data regarding effective treatments for oligoarticular disease and the inception point between oligoarticular and polyarticular disease are sparse.

Apremilast is an oral phosphodiesterase-4 inhibitor approved for PsA, psoriasis (PsO), palmoplantar pustulosis and Behçet’s disease [13, 14]. FOREMOST (NCT03747939) is the first randomized, placebo-controlled trial in early (mean duration, 9.9 months) oligoarticular PsA and demonstrated the efficacy of apremilast *vs* placebo over 16 weeks [13]. Of note, 35% of patients randomized to placebo progressed from ≤4 active (swollen and/or tender) joints at baseline to >4 active joints at week 16, compared with 20% randomized to apremilast, suggesting apremilast delays or prevents polyarticular progression. Using FOREMOST data, our objectives were to: (i) identify predictors of progression from oligoarticular to polyarticular PsA; (ii) describe joints involved in early oligoarticular PsA; and (iii) report efficacy and tolerability of apremilast treatment for up to 48 weeks in patients with early PsA.

## Methods

### Study design and patient population

FOREMOST (ClinicalTrials.gov identifier: NCT03747939) has been described previously [13]. Patients with early PsA (duration ≤5 years) and limited joint involvement (>1 to ≤4 swollen joint count [SJC] and >1 to ≤4 tender joint count [TJC]; 66–68 joints assessed), despite treatment with non-steroidal anti-inflammatory drugs (NSAIDs) and/or conventional synthetic disease-modifying antirheumatic drugs (csDMARDs), were randomized 2:1 to apremilast or placebo for 24 weeks [13] (Fig. S1). At week 16, patients with no improvement in SJC were eligible for early escape: patients randomized to placebo could transition to apremilast, patients randomized to apremilast continued apremilast. From week 24, all patients could receive open-label apremilast through week 48. Concomitant csDMARDs (methotrexate or sulfasalazine), NSAIDs and low dose corticosteroids were permitted throughout FOREMOST (stable dose during placebo-controlled period) and considered standard of care.

FOREMOST was approved by institutional review boards/ethics committees at each site and conducted in compliance with Good Clinical Practice Guidelines, the International Council for Harmonisation Guideline E6, the Declaration of Helsinki, and applicable regulatory requirements [13]. Patients provided written informed consent before study-related procedures.

### Outcomes

We report *post hoc* analyses (proposed by the FOREMOST study Steering Committee) of baseline joint involvement and disease progression, including predictors of progression. Progression was defined as moving from oligoarticular (≤4 active joints) PsA at baseline (enrolment) to polyarticular (>4 active joints) PsA post-baseline. We also report pre-specified endpoints (listed in Table S1) through week 48.

### Statistical analysis

#### Baseline joint involvement

Baseline patterns of active joints in overall FOREMOST population were summarized for 10 joint groups: (i) temporomandibular and shoulder (*n *= 8 individual small [temporomandibular] and large [shoulder] joints); (ii) elbow (*n *= 2 intermediate); (iii) wrist (*n *= 2 small); (iv) metacarpophalangeal (MCP; *n *= 10 small); (v) finger distal interphalangeal (finger DIP; *n *= 8 small); (vi) finger proximal interphalangeal (finger PIP; *n *= 10 small); (vii) knee (*n *= 2 large); (viii) ankle and tarsus/midfoot (*n *= 4 large [ankle] and small [tarsus/midfoot]); (ix) metatarsophalangeal (MTP; *n *= 10 small); (x) toe PIP (*n *= 10 small). For each group, the number (%) of patients with at least one active joint was summarized overall and by sex.

#### Predictors of disease progression at week 16

As a first step, subgroup analyses assessed progression rates (and treatment differences [apremilast *vs* placebo]) at week 16 by baseline patient/disease characteristics. Multivariable logistic regression then modelled predictors of progression, with covariates chosen based on the subgroup analyses and clinical relevance (Fig. S2) and last observation carried forward (LOCF) used to impute missing joint assessments. Backward selection (*P<*0.05) identified statistically significant predictors; based on clinical relevance [11], body mass index (BMI) and dactylitis (measured using the Leeds Dactylitis Index [LDI]) were forced to remain in the model. Odds ratios (OR) with 95% confidence interval (CIs) are reported for covariates remaining in the final model.

To assess the role of apremilast as a disease modifier and maximize statistical power, the above analyses were initially performed using the overall FOREMOST population. To explore trends without treatment effect, they were repeated using data from patients randomized to placebo through week 24.

#### Pre-specified endpoints through week 48

Disease activity goals, signs and symptoms were analyzed through week 48 in patients receiving at least one apremilast dose. ‘APR’ denotes data through week 48 for patients receiving apremilast from randomization. For patients transitioning from placebo to apremilast at week 16 or 24, ‘PBO’ denotes data from baseline to week 16 and ‘PBO/APR’ denotes data after week 16. Endpoints were summarized using descriptive statistics with data reported as observed. Analysis of joint endpoints was based on all joints. Subgroup analysis assessed endpoints through week 48 in csDMARD-experienced patients.

## Results

### Disease progression and predictors of progression

**Figure 1:**
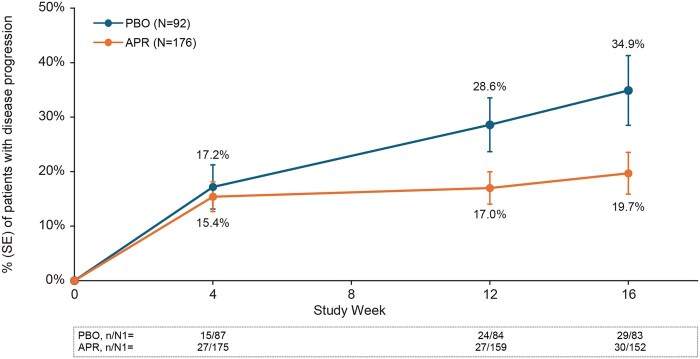
Progression from oligoarticular to polyarticular PsA through week 16 in the overall FOREMOST population. Data reported as observed for *n*=268 randomized patients with ≤4 active (swollen and/or tender) joints at baseline. Progression from oligoarticular to polyarticular PsA defined as moving from ≤4 active joints at baseline to >4 active joints post-baseline. APR, apremilast; n, number of patients with disease progression at each time point; N1, number of patients with non-missing data at each time point; PBO, placebo; PsA, psoriatic arthritis; SE, standard error

**Figure 2:**
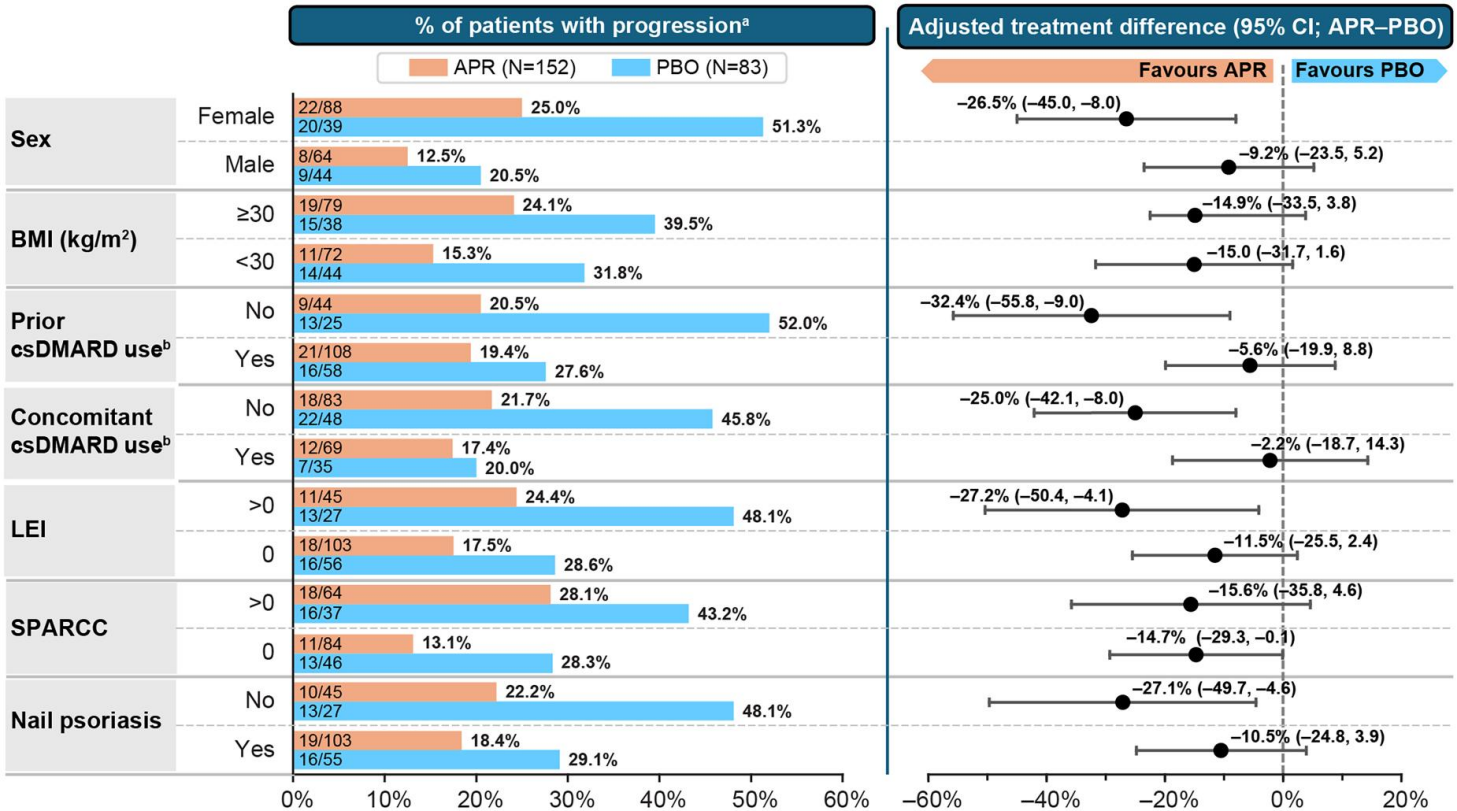
Subgroup analysis of progression from oligoarticular to polyarticular PsA at week 16 of FOREMOST. Data reported as observed for *n *= 235 randomized patients with ≤4 active (tender and/or swollen) joints at baseline and non-missing joint data at week 16. Progression from oligoarticular to polyarticular PsA defined as moving from ≤4 active joints at baseline to >4 active joints post-baseline. ^a^Numerator/denominator for each % shown on figure. ^b^Prior csDMARD use = prior to enrolment into FOREMOST; concomitant csDMARD use = stable methotrexate or sulfasalazine through Week 16. APR, apremilast; BMI, body mass index; CI, confidence interval; csDMARD, conventional systemic antirheumatic drug (methotrexate or sulfasalazine); LEI, Leeds Enthesitis Index; PBO, placebo; PsA, psoriatic arthritis; SPARCC, Spondyloarthritis Research Consortium of Canada

Most (268/308 [87.0%]) patients in the overall FOREMOST population had ≤4 active joints at baseline. Of these, 25.1% (59/235; data as observed) progressed to >4 active joints by week 16, with lower rates of progression for apremilast *vs* placebo (19.7% *vs* 34.9%; Fig. 1) and this trend consistent across subgroups (Fig. 2). In the apremilast group, disease progression mostly occurred by week 4; patients receiving placebo continued to progress through week 16. Greater treatment differences were observed in females *vs* males (–26.5% [95% CI, –45.0, –8.0] *vs* –9.2% [–23.5, 5.2]), csDMARD-naive *vs* csDMARD-experienced patients (–32.4% [–55.8, –9.0] *vs* –5.6% [–19.9, 8.8]), patients without *vs* with concomitant (on-study) csDMARD use (–25.0% [–42.1, –8.0] *vs* –2.2% [–18.7, 14.3]), patients with *vs* without enthesitis measured using the Leeds Enthesitis Index (LEI; LEI > 0, –27.2% [–50.4, –4.1] *vs* LEI = 0, –11.5% [–25.5, 2.4]), and patients without *vs* with nail PsO (–27.1% [–49.7, –4.6] *vs* –10.5% [–24.8, 3.9]). Treatment differences were consistent regardless of baseline BMI (<30 *vs* ≥30 kg/m^2^) and when enthesitis was measured using the Spondylarthritis Research Consortium of Canada (SPARCC) Index.

**Figure 3:**
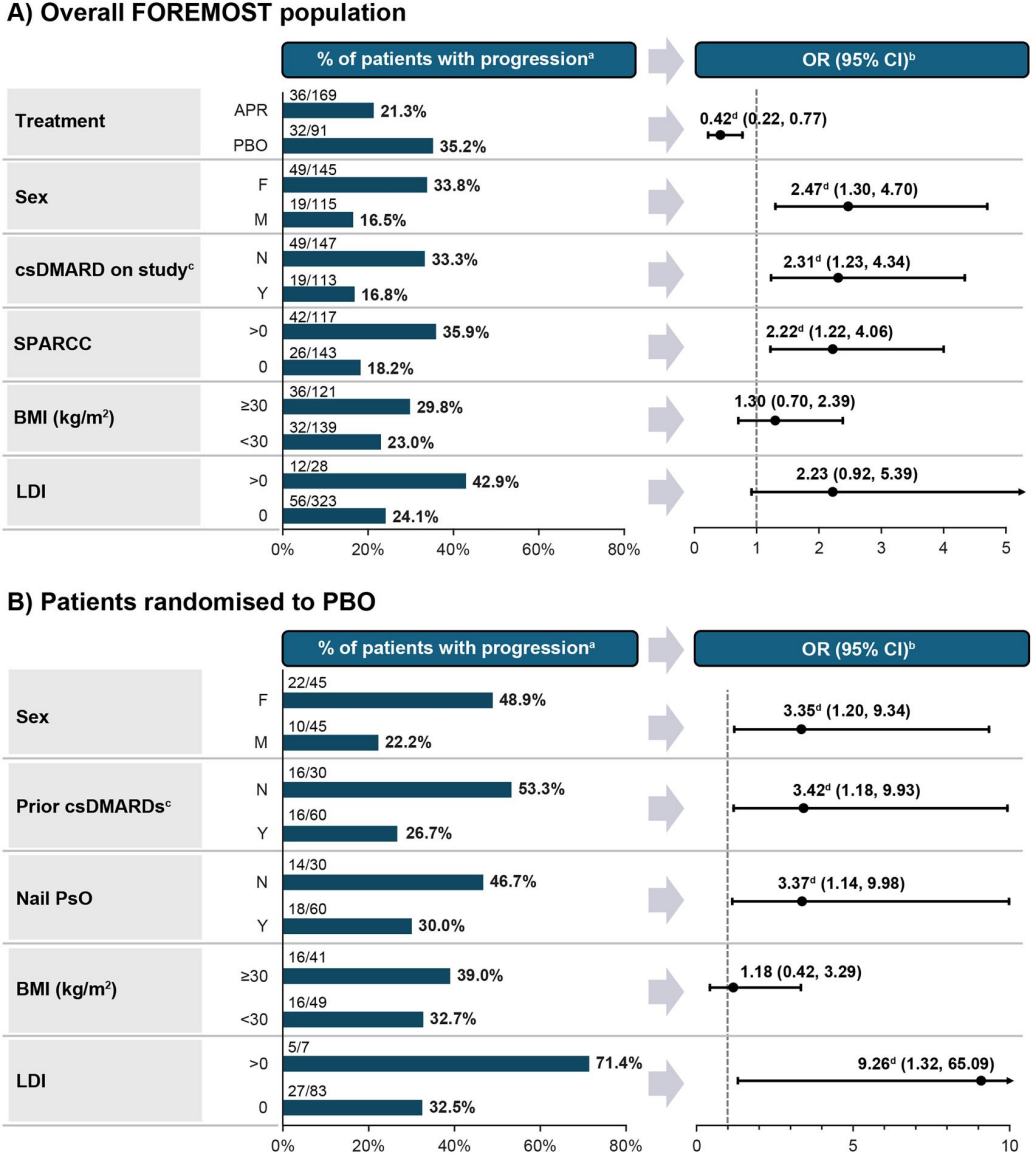
Multivariable logistic regression of progression from oligoarticular to polyarticular PsA at week 16 of FOREMOST. Data reported for (**A**) *n*=260 patients with ≤4 active (swollen and/or tender) joints at baseline and non-missing covariates in the final model; (**B**) *n* = 90 patients randomized to PBO with ≤4 active joints at baseline and non-missing covariates in the final model. Missing joint data at Week 16 imputed using LOCF. Progression from oligoarticular to polyarticular PsA defined as moving from ≤4 active joints at baseline to >4 active joints post-baseline. ^a^Numerator/denominator for each % shown on figure. ^b^OR for first vs second category. ^c^Prior csDMARD use = prior to enrolment into FOREMOST; csDMARDS on study = stable methotrexate or sulfasalazine through week 16. ^d^Indicates ORs for which the 95% CIs do not include 1. APR, apremilast; BMI, body mass index; CI, confidence interval; csDMARD, conventional systemic antirheumatic drugs (stable methotrexate or sulfasalazine); F, female; LDI, Leeds Dactylitis Index; LOCF, last observation carried forward; M, male; N, no; OR, odds ratio; PBO, placebo; PsA, psoriatic arthritis; PsO, psoriasis; SPARCC, Spondyloarthritis Research Consortium of Canada; Y, yes

In the overall FOREMOST population (Fig. 3A; *N* = 260 patients in final model), apremilast was associated with statistically significant lower odds of progression at week 16 *vs* placebo (OR [95% CI]: 0.42 [0.22, 0.77]; relative risk [RR]: 21.3% *vs* 35.2%). After adjusting for treatment effect, being female (*vs* male; OR [95% CI]: 2.47 [1.30, 4.70]; RR: 33.8% *vs* 16.5%), not receiving (*vs* receiving) concomitant csDMARDs (2.31 [1.23, 4.34]; 33.3% *vs* 16.8%) and having active enthesitis as measured using the SPARCC Index (SPARCC > 0 *vs* SPARCC = 0; 2.22 [1.22, 4.06]; 35.9% *vs* 18.2%) significantly increased the odds of progression. A non-significant trend towards higher odds of progression was observed for patients with *vs* without dactylitis measured using the Leeds Dactylitis Index (LDI; LDI > 0 *vs* LDI = 0; OR [95% CI]: 2.23 [0.92, 5.39]; RR: 42.9% *vs* 24.1%).

Limiting the multivariate analysis to patients receiving placebo through week 16 (Fig. 3B; *N* = 90 patients in final model), being female (*vs* male; OR [95% CI]: 3.35 [1.20, 9.34]; RR, 48.9% *vs* 22.2%), being csDMARD naive (*vs* csDMARD-experienced; 3.42 [1.18, 9.93]; 53.3% *vs* 26.7%), having dactylitis (LDI > 0 *vs* LDI = 0; 9.26 [1.32, 65.09]; 71.4% *vs* 32.5%) and the absence of nail PsO (*vs* presence of nail PsO; 3.37 [1.14, 9.98]; 46.7% *vs* 30.0%) significantly increased the odds of progression. BMI (categorized as <30 *vs* ≥30 kg/m^2^) did not predict disease progression in the overall study population or placebo data. Enthesitis (measured by LEI or SPARCC Index) was not a predictor of progression in the placebo data.

**Figure 4:**
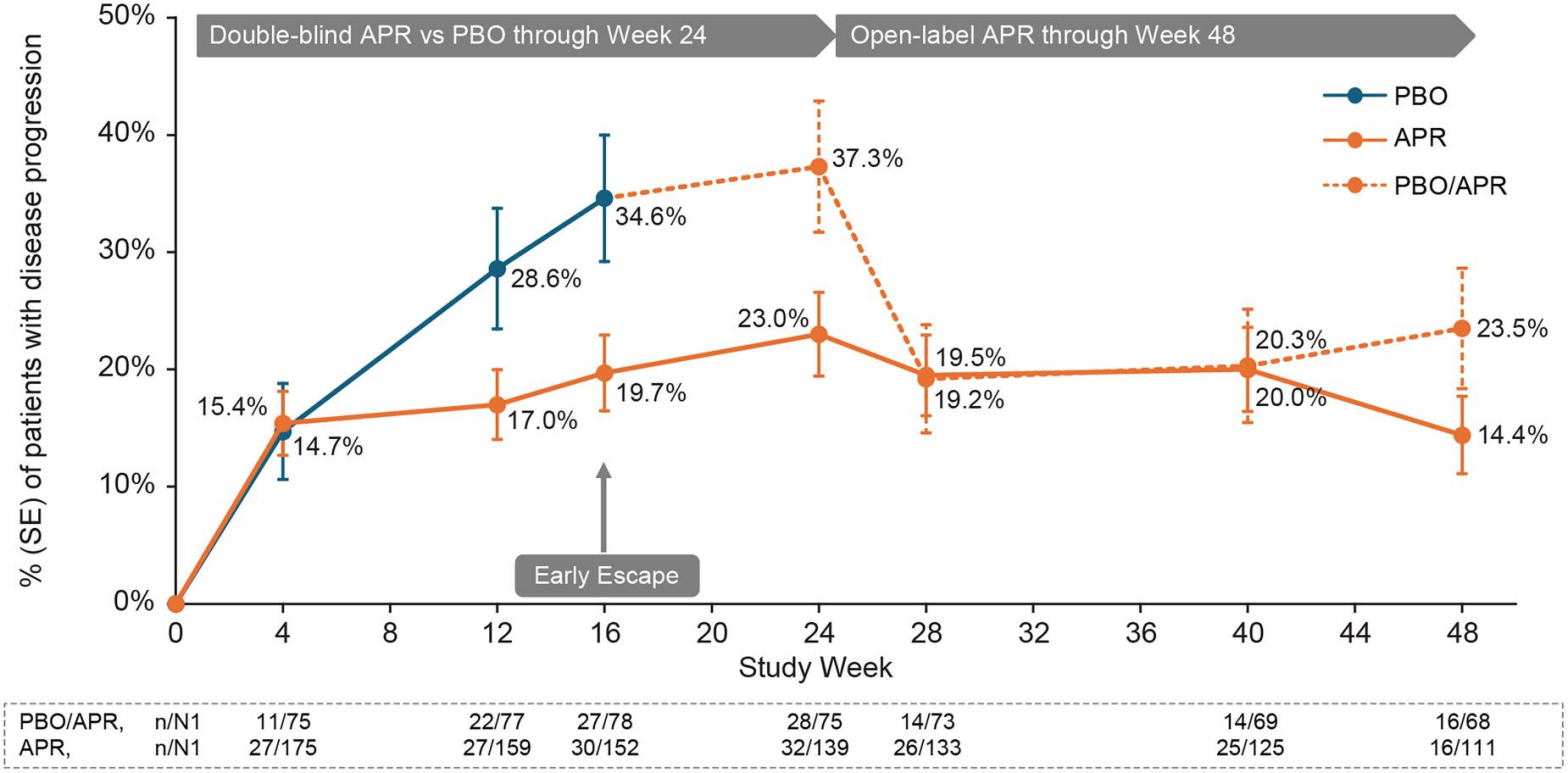
Progression from oligoarticular to polyarticular PsA in patients receiving ≥1 apremilast dose in FOREMOST. Data reported as observed for *n*=254 patients with ≤4 active (swollen and/or tender) joints at baseline receiving at least one dose of APR. ‘APR’ denotes data through week 48 for patients receiving APR from randomization. For patients transitioning from PBO to APR, ‘PBO’ denotes data from baseline to Week 16 and ‘PBO/APR’ denotes data after week 16. Progression from oligoarticular to polyarticular PsA defined as moving from ≤4 active joints at baseline to >4 active joints post-baseline. APR, apremilast; n, number of patients with disease progression at each time point; N1, number of patients with non-missing data at each timepoint; PBO, placebo; PsA, psoriatic arthritis; SE, standard error

Among patients receiving at least one apremilast dose in FOREMOST (as randomized or switched from placebo; *N* = 291), 17.9% (32/179) of patients had progressed from ≤4 active joints at baseline to >4 active joints at week 48, with fewer placebo patients experiencing progression after switching to apremilast [23.5% at week 48 (after switching) *vs* 34.6% at week 16 (pre switch); Fig. 4]. Mean TJC and active joint count increased on placebo (baseline to week 16) and decreased when patients switched to apremilast through week 48 (Fig. S3). Mean SJC decreased through week 48, with larger decreases observed for apremilast *vs* placebo through week 16, and the decreases observed with apremilast at week 16 maintained through week 48.

### Patterns of joint involvement

**Figure 5:**
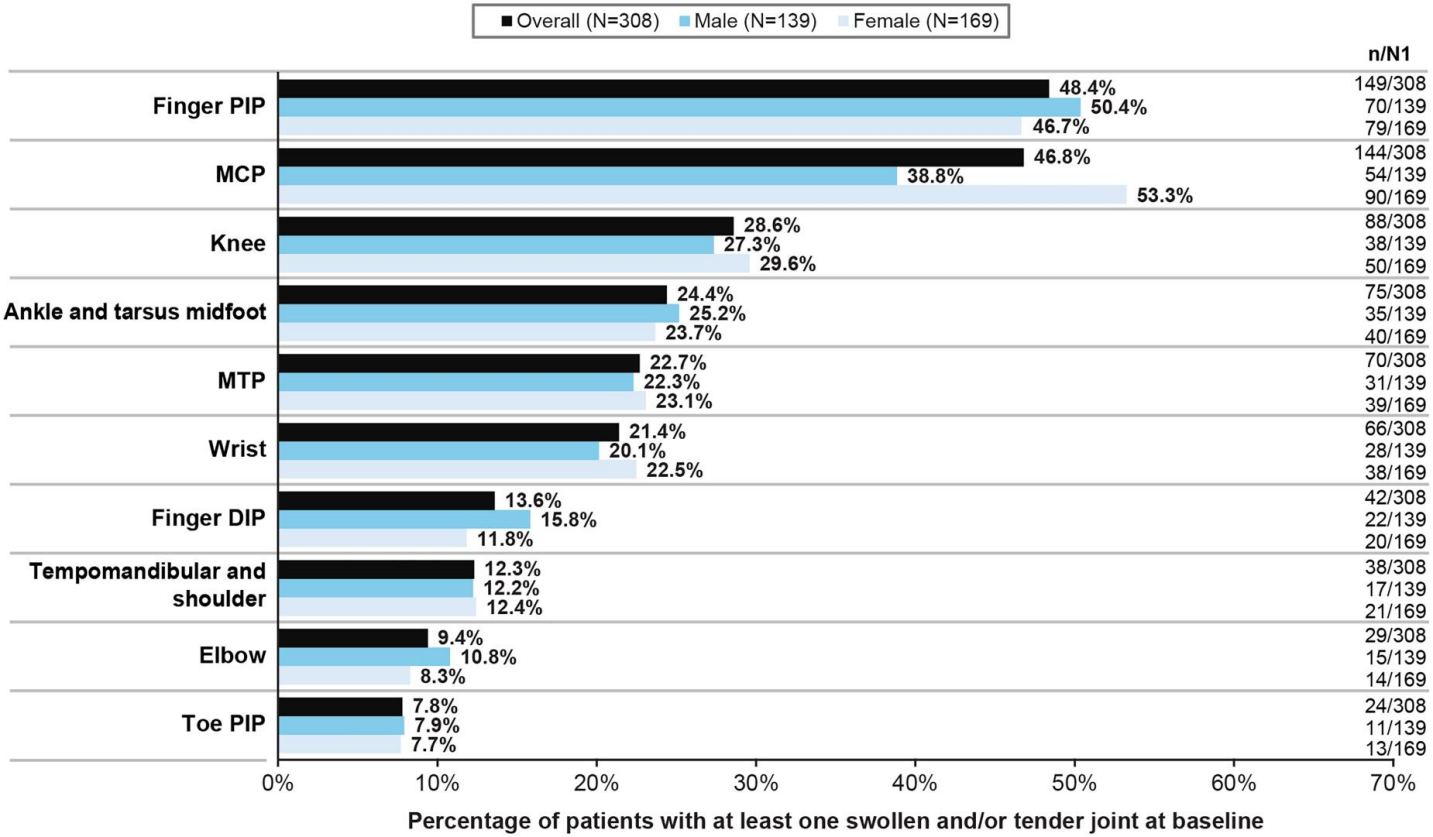
Baseline patterns of active joints in the overall FOREMOST study population. Data reported as observed for N=308 randomized patients. DIP, distal interphalangeal; MCP, metatarsophalangeal; MTP, metatarsophalangeal; n, number of patients with ≥1 active (swollen and/or tender) joint in the joint group; N1, number of patients with non-missing data for the joint group; PIP, proximal interphalangeal

In the overall FOREMOST population (*N* = 308), finger PIP, MCP, knee and ankle and tarsus/midfoot joints were most likely to be involved at baseline: 48.4%, 46.8%, 28.6% and 24.4% of patients with ≥1 active joint at baseline, respectively (Fig. 5). Females had higher baseline incidence of MCP joints than males (53.3% *vs* 38.8%). Other baseline characteristics were similar for males and females (Table S2).

### Disease activity goals and improvements in disease signs and symptoms with up to 48 weeks of apremilast treatment

Among *N* = 291 patients receiving at least one apremilast dose (*n* = 203 from randomization; *n* = 88 transitioned from placebo), most (210/291 [72.2%]) completed the open-label extension phase through week 48; one PBO patient transitioning to APR at week 16 did not enter the extension phase (Fig. S4). Reasons for apremilast discontinuation were: adverse event (9.2%); lack of efficacy (6.2%); patient withdrawal (5.2%); lost to follow-up (2.7%); noncompliance with study drug, death and pregnancy (0.3% each); and ‘other’ (3.1%).

Baseline characteristics of patients receiving at least one apremilast dose (Table S3 and S4) were similar to those of the overall study population [13]. Most (73.2%; PBO/APR, 68.2%; APR, 75.4%) patients had clinical Disease Activity in Psoriatic Arthritis (cDAPSA) moderate disease activity (ModDA) at baseline; mean (SD) cDAPSA score, 16.1 (4.4). Mean (SD) baseline Routine Assessment of Patient Index (RAPID3) score was 13.47 (5.36) (PBO/APR, 13.07 [5.41]; APR, 13.65 [5.35]).

**Figure 6:**
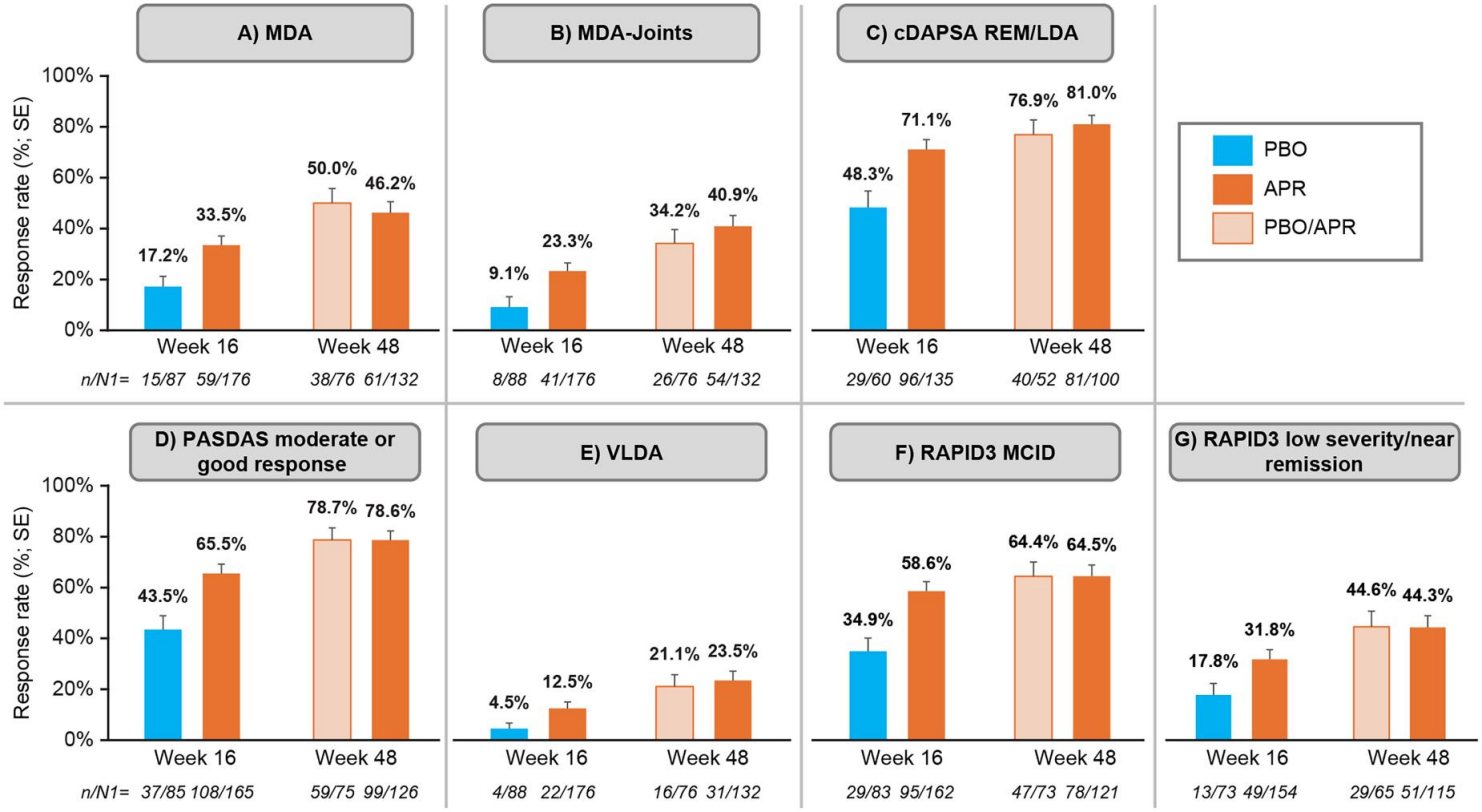
Achievement of disease activity goals in patients receiving ≥1 apremilast dose in FOREMOST. (**A**, **B**, **D** and **E**) report data as observed for *n *= 291 patients receiving at least one dose of APR. (**C**) Reports data as observed for *n *= 213 patients with baseline cDAPSA High or Moderate Disease Activity receiving at least one dose of APR. (**F**) Reports data as observed for *n *= 272 patients with baseline RAPID3 score **≥**3.8 receiving at least one dose of APR. (**G**) Reports data as observed for *n* = 253 patients with baseline RAPID3 score >6 (high/moderate severity) receiving at least one dose of APR. APR denotes week 16 and week 48 data in patients receiving APR from randomization. PBO denotes Week 16 data in patients randomized to receive PBO through week 24. PBO/APR denotes week 48 data in patients randomized to PBO who transitioned to APR at week 16 or 24. APR, apremilast; cDAPSA, Clinical Disease activity in Psoriatic Arthritis; LDA, low disease activity; MCID, Minimal Clinically Important Difference; MDA, minimal disease activity; MDA-Joints, Modified MDA; n, number of patients achieving disease activity goal; N1, number of patients with non-missing data; PASDAS, Psoriatic Arthritis Disease Activity Score; PBO, placebo; RAPID3, Routine Assessment of Patient Index; REM, remission; SE, standard error; VLDA, very low disease activity

Among patients receiving apremilast from randomization, 46.2% achieved Minimal Disease Activity (MDA) at week 48, with a similar response rate in placebo patients switching to apremilast (PBO/APR, 50.0%), and a similar trend observed for MDA-Joints (Fig. 6A and B). Among patients with cDAPSA ModDA at baseline, 81.0% receiving apremilast from randomization moved to low disease activity or remission (LDA/REM) at week 48, with a similarly high response rate in placebo patients switching to apremilast (PBO/APR; 76.9%) (Fig. 6C). A total of 23.5% of patients receiving apremilast from randomization achieved very low disease activity (VLDA) at week 48, with a similar response rate in placebo patients switching to apremilast (PBO/APR, 21.1%) (Fig. 6E). Regardless of when apremilast was initiated, 79% (APR, 78.6%; PBO/APR, 78.7%) of patients achieved Psoriatic Arthritis Disease Activity Score (PASDAS) moderate or good response at week 48 (Fig. 6D). Approximately two-thirds (APR, 64.5%; PBO/APR, 64.4%) of patients with a baseline RAPID3 score **≥**3.8 achieved RAPID3 minimal clinically important difference (MCID) at week 48 (Fig. 6F) and 44% (APR, 44.3%; PBO/APR, 44.6%) with RAPID3 moderate or high severity at baseline achieved low severity or near remission at week 48 (Fig. 6G).

One-third (34.4% [100/291]) of patients receiving at least one apremilast dose had an LEI score >0 at baseline (APR, 34.5% [70/203]; PBO/APR, 34.1% [30/88]), of whom approximately two-thirds (APR, 63.3%; PBO/APR, 66.7%) achieved an LEI score of 0 at week 48; mean change from baseline, –1.6 (PBO/APR; SE, 0.23) to –1.7 (APR; SE, 0.52) (Fig. S5A and B). A total of 47.1% (137/291) of patients receiving at least one apremilast dose had SPARCC score >0 at baseline (APR, 46.3% [94/203]; PBO/APR, 48.9% [43/88]), of whom over half (APR, 52.3%; PBO/APR, 61.1%) achieved a SPARCC score of 0 at week 48 (Fig. S5C).

Fig. S6 summarizes improvements in Physician’s Global Assessment (PhGA) and Patient Global Assessment (PtGA) through week 48, with mean (SE) changes of –29.8 (1.80) (APR) to –28.1 (2.90) (PBO/APR) mm and –21.8 (2.67) (APR) to –21.1 (1.82) (PBO/APR) mm, respectively, between baseline and week 48.

### Efficacy of apremilast in csDMARD-experienced patients

Baseline disease characteristics/disease activity and apremilast efficacy in csDMARD-experienced patients were consistent with those observed for all patients receiving at least one apremilast dose through week 48 (Supplementary Tables S3 and S5; Supplementary Figs S7 and S8).

### Treatment emergent adverse events during apremilast exposure

Among patients receiving at least one apremilast dose (*N* = 291; 192.5 patient-years of apremilast exposure), 63.6% (185/291) reported at least one TEAE during apremilast treatment; exposure-adjusted incidence rate, 184.1 per 100 patient-years (Table S5). Two deaths occurred in patients receiving apremilast during the placebo-controlled phase (assessed by study investigators as not related to study drug [13]); no deaths occurred during the open-label extension. The safety profile of apremilast through week 48 was similar to that observed through week 16 (Table S5) and week 24 [13], and consistent with the established safety profile of apremilast.

## Discussion

We report previously unexplored aspects of early oligoarticular PsA, which could inform clinical practice, including predictors of progression to polyarticular disease, patterns of joint involvement and the role of apremilast as a disease modifier. We found both large (knee, ankle) and small (finger PIP, MCP, tarsus/midfoot) joints contributed to early oligoarticular PsA, and being female, being csDMARD-naive and having dactylitis predicted polyarticular progression. Furthermore, apremilast halved the odds of polyarticular progression, with treatment benefits maintained through 48 weeks.

These insights are gleaned from FOREMOST, in which most (87%) patients had ≤4 active joints at baseline and mean (SD) disease duration was 9.9 (10.2) months, indicating early oligoarticular PsA. With both large and small joints involved at this early disease stage, our data indicate clinicians should make a complete examination of all joints (large and small) in patients presenting with suspected or early oligoarticular PsA. Finger PIP, MCP and knee joints were most likely to be involved and, adding to a growing body of evidence on the role of sex in PsA [15, 16], females were more likely to have disease involvement at MCP joints than males. Overall, one-quarter (25.1%) of patients with ≤4 active joints at baseline progressed to >4 active joints by week 16, with lower rates of progression for patients receiving apremilast than placebo (19.7% *vs* 34.9%). This low rate of disease progression and improvements in disease activity and clinical signs and symptoms were maintained with up to 48 weeks of apremilast treatment, and no new safety signals were observed. Among patients with disease progression, those receiving apremilast typically experienced progression within 4 weeks of treatment initiation, suggesting rapid disease control. In contrast, patients receiving placebo continued to experience progression through week 16. Considering lack of efficacy data regarding other treatments in early oligoarticular PsA, these findings are of high interest and could inform treatment decisions.

When modelling predictors of disease progression, we used the overall FOREMOST population to maximize statistical power and assess the potential role of apremilast as a disease modifier. Compared with placebo ± standard of care (stable NSAIDs and/or csDMARDs), apremilast more than halved the odds of progressing from oligoarticular to polyarticular disease at week 16. After adjusting for treatment effect, being female, not receiving concomitant csDMARDs and presence of enthesitis were statistically significant predictors, more than doubling the odds of disease progression. With entheseal disease assessed clinically and not confirmed by imaging [17], findings regarding enthesitis should be interpreted with caution.

To explore trends without the effect of apremilast, these analyses were repeated using data from patients receiving placebo ± standard of care through week 16, with the presence of dactylitis increasing odds of progression approximately nine-fold and being female and csDMARD-naïve each increasing the odds more than three-fold. While our results should be interpreted with caution due to small sample sizes in some subgroups, they provide novel insights on the oligoarticular PsA phenotype, including risk factors for progression to polyarticular disease. Such data are currently sparse and recognized as a key research area [11].

While over two-thirds (69%) of patients in FOREMOST had nail PsO, and despite guidelines recommending csDMARDs in patients with poor prognostic factors, including nail involvement [18], one-third were csDMARD-naïve and fewer than half received concomitant csDMARDs on study. With progression to polyarticular disease associated with joint damage [9–11], closing this treatment gap is critical to improve patient outcomes and FOREMOST demonstrates the clinical value of apremilast in this setting. The open-label MOSAIC study of patients with PsA, hand involvement and prior csDMARD use provides further evidence of apremilast’s clinical value, with MRI assessments showing improvements in joint damage after 24 and 48 weeks of treatment [19].

EULAR and GRAPPA guidelines recommend disease remission or low disease activity as a treatment target for PsA [18, 20]. In FOREMOST, over three-quarters (77–81%) of patients receiving apremilast for up to 48 weeks achieved cDAPSA LDA/REM or PASDAS moderate/good response, at least one-fifth (21–24%) achieved VLDA, and approximately three-fifths (64–65%) achieved RAPID3 MCID. With these disease activity goals capturing patient-perceived disease burden, our data demonstrate apremilast’s sustained benefits in early oligoarticular PsA, including a reduction in disease burden. We also observed sustained improvements in tender and swollen joints, and enthesitis, clinical manifestations which are included in the GRAPPA domain-based recommendations [20]. Despite the potential for reporting bias when receiving open-label *vs* blinded apremilast, TEAE exposure-adjusted incident rates through week 48 were lower than observed through week 16 (184.1 *vs* 277.5 per 100 patient-years), and tolerability remained consistent with the known apremilast safety profile.

FOREMOST can be compared with several real-world cohorts of early (<12 months) oligoarticular/peripheral PsA. The University of Toronto cohort reported a mix of small and large joint involvement, most commonly hand, knee and feet joints, with 14% of patients (*N* = 192) receiving baseline csDMARDs and 39% progressing to polyarticular disease at 9–10 years follow-up, compared with 40% baseline csDMARD use and 14.4% to 23.5% progression at week 48 of FOREMOST [2]. The first 186 patients enrolled in the APACHE cohort had similar baseline csDMARD use and cDAPSA score to FOREMOST (38% *vs* 40% and 19.0 *vs* 16.2, respectively) [7]. In an Irish cohort of *N* = 129 patients, 12% were receiving baseline csDMARDs, with increased DMARD use (59% and 56%) at one and two years follow-up, corresponding disease remission rates of 26% and 21%, respectively, and no association between disease progression and sex, enthesitis or csDMARD use [5]. In data from the National Early Inflammatory Arthritis Audit (May 2018 to October 2019), and compared with patients with rheumatoid arthritis, patients with PsA experienced longer delays between symptoms and referrals, and between presentation to a primary physician and diagnosis [12]. With FOREMOST, and despite the emphasis of EULAR recommendations on earlier and more aggressive treatment [18], these studies provide further evidence of undertreatment of early oligoarticular PsA and highlight a window of opportunity to attenuate disease progression in patients with early PsA. Furthermore, they demonstrate the benefit of initiating apremilast early in the treatment paradigm, including before conventional csDMARDs.

Collectively, endpoints used in FOREMOST reflect the heterogeneity of early oligoarticular PsA, capturing clinical manifestations and patient-perceived disease burden. Significant improvements were observed with apremilast *vs* placebo across multiple measures of disease activity and maintained or improved with up to 48 weeks of treatment. Limitations of our data include the *post hoc* nature of some analyses and small sample sizes for patient subgroups. In addition, with mean disease duration in FOREMOST only 9.9 months, and patients with more severe disease more likely to agree to participate in clinical trials, FOREMOST may not be representative of all patients with oligoarticular PsA. Oligoarticular disease was identified using clinical joints alone and imaging was not used to exclude subclinical synovitis; therefore, some patients categorized as oligoarticular may have had subclinical involvement of additional joints. In these patients, the observed progression to polyarticular disease could reflect the clinical emergence of existing subclinical inflammation rather than true progression. While nail PsO is a known risk factor for PsA [21–23], nail PsO decreased odds of progressing from oligoarticular to polyarticular PsA. Due to small sample size, this could be a spurious result and should be interpreted with caution. In addition, this association was only observed for patients receiving placebo and was not seen in the overall FOREMOST population.

In conclusion, data from FOREMOST suggest both large and small joints contribute to early oligoarticular PsA, with being female, naive to csDMARDs and having dactylitis potential predictors of polyarticular progression. Moreover, our data demonstrate initiating treatment during the early, oligoarticular phase of PsA, as recommended by EULAR [18, 24], reduces disease activity and rapidly delays/attenuates progression to polyarticular disease, and highlight the benefits of apremilast, which reduced the odds of progression by 58%. Our findings should be confirmed in other datasets, and their implications for real-world clinical practice assessed.

## Supplementary Material

keag351_Supplementary_Data

## Data Availability

Qualified researchers may request data from Amgen clinical studies. Complete details are available at http://www.amgen.com/datasharing.
