# Supplementary material for: Progression from oligoarticular to polyarticular psoriatic arthritis and apremilast as a disease modifier: Novel insights from FOREMOST
Source: Rheumatology (Oxford). 2026 Jul 1;65(7):keag351. doi: 10.1093/rheumatology/keag351 (PMC13387600; doi:10.1093/rheumatology/keag351)
Supplement: keag351_Supplementary_Data [file keag351_supplementary_data.pdf]

Supplementary Material

Progression from oligoarticular to polyarticular psoriatic arthritis and apremilast as a disease modifier: Novel insights from FOREMOST

Authors: Laura C. Coates[1], Dafna D. Gladman[2], Joseph F. Merola[3], Ulrich Mrowietz[4], April Armstrong[5], Xenofon Baraliakos[6], William Tillett[7], Mitsumasa Kishimoto[8], Jyotsna Reddy[9], Lichen Teng[9], Hamid Amouzadeh[9], Cynthia Deignan[9], Philip J. Mease[10], Laure Gossec[11,12]

Author Institutions:

[1]Nuffield Department of Orthopaedics, Rheumatology and Musculoskeletal Sciences, University of Oxford, Oxford, UK

[2]Schroeder Arthritis Institute, Krembil Research Institute, Toronto Western Hospital, Department of Medicine, Rheumatology, University of Toronto, Toronto, Ontario, Canada

[3]Department of Dermatology and Department of Medicine, Division of Rheumatology, UT Southwestern Medical Center, Dallas, Texas, USA

[4]Psoriasis Center, Department of Dermatology, University Medical Center Schleswig-Holstein, Kiel, Germany

[5]Dermatology, University of California Los Angeles, Los Angeles, CA, USA

[6]Rheumazentrum Ruhrgebiet, Herne, Germany and Ruhr-University Bochum, Germany

[7]Department of Life Sciences, University of Bath, Bath, UK

[8]Department of Nephrology and Rheumatology, Kyorin University School of Medicine, Tokyo, Japan

[9]Amgen Inc, Thousand Oaks, California, USA

[10]Swedish Medical Center/Providence St. Joseph Health and University of Washington School of Medicine, Seattle, Washington, USA

[11]Sorbonne Université, INSERM, Institut Pierre Louis d'Epidémiologie et de Santé Publique, Paris, France

[12]Rheumatology Department, AP-HP, Pitié Salpêtrière Hospital, Paris, France

Target journal: Rheumatology (Oxford)

Supplementary material: 5 tables, 8 figures

Supplementary Table S1. FOREMOST study outcomes, A) Disease activity goals, B) Clinical and patient-reported outcomes, C) Tolerability

A) Disease activity Goals

**cDAPSA [1, 2]**	• Clinical Disease Activity in Psoriatic Arthritis<br>• Composite measure of disease activity<br>• Includes four disease activity domains<br>1. tender joints<br>2. swollen joints<br>3. patient global assessment of disease activity (PtGA, based on 10 cm visual analogue scale [VAS])<br>4. patient pain VAS (based on 10 cm scale)<br>• Range, 0-154; higher scores indicate greater disease severity; set to missing if any domain is missing<br>– Remission (REM): score ≤4<br>– Low disease activity (LDA): score >4 to ≤13<br>– Moderate disease activity (ModDA): score >13 to ≤27<br>– High disease activity (HDA): score >27
**MDA [3-5]**	• Minimal Disease Activity<br>• Defined as achieving 5 of the following domains:<br>1. Swollen Joint Count (SJC; 0-66) ≤1<br>2. Tender Joint Count (TJC; 0-68) ≤1<br>3. Psoriasis Body Surface Area (BSA) ≤3%<br>4. Patient-reported pain VAS ≤15 mm (based on 100 mm scale)<br>5. PtGA ≤20 mm (based on 100 mm scale)<br>6. Patient-reported Health Assessment Questionnaire Disability Index (HAQ-DI) ≤0.5<br>**7.** Leeds Enthesitis Index (LEI) ≤1
**MDA-Joints [6]**	• Modified MDA<br>• Modified disease activity goal with a focus on improvement in the number of active joints<br>• Defined as achieving both SJC (0-66) ≤1 and TJC (0-68) ≤1, and ≥3 of the following criteria:<br>1. Psoriasis BSA ≤3%<br>2. Patient pain VAS ≤15 mm (based on 100 mm scale)

	3. PtGA ≤20 mm (based on 100 mm scale)<br>4. HAQ-DI ≤0.5<br>5. LEI ≤1
**PASDAS**	• Psoriatic Arthritis Disease Activity Score<br>• Weighted index comprising assessments of joints, function, acute-phase response, quality of life (QoL), PtGA and physician global assessment of disease (PGA), enthesitis and dactylitis; set to missing if at least one component is missing<br>• Range, 0-10; higher scores indicate worse disease activity<br>– Good response: final score ≤3.2 and ≥1.6 improvement from baseline<br>– Moderate response: final score ≤3.2 and >0.8 to <1.6 improvement, or final score >3.2 to <5.4 and >0.8 improvement, or final score ≥5.4 and ≥1.6 improvement<br>– Poor response: final score ≥5.4 and >0.8 to <1.6 improvement, or ≤0.8 improvement
**RAPID3** [7, 8]	• Routine Assessment of Patient Index Data<br>• Composite of 3 patient-reported outcomes:<br>1. HAQ-DI<br>2. Pain VAS<br>3. PtGA<br>• Range, 0 to 30<br>– Near remission: score ≤3<br>– Low disease severity score: >3 to ≤6<br>– Moderate severity: score >6 to ≤12<br>– High severity: score >12<br>– Minimal clinically important difference (MCID): ≥3.8 improvement from baseline
**VLDA** [4]	• Very Low Disease Activity<br>• Defined as achieving all 7 domains of MDA

B) Clinical and patient-reported outcomes

**Disease progression**	• Progression from oligoarticular to polyarticular disease<br>• Defined as moving from ≤4 active (tender and/or swollen) joints at baseline to >4 active joints at/by selected timepoint
**LDI**	• Leeds Dactylitis Index<br>• Range, 0 to 60; higher values indicate more severe dactylitis
**LEI**	• Leeds Enthesitis Index<br>• Range, 0 to 6; higher values indicate higher levels of enthesitis
**SPARCC**	• Spondyloarthritis Research Consortium of Canada Index for enthesitis<br>• Range, 0 to 16; higher values indicate higher levels of enthesitis
**PhGA**	• Physician's Global Assessment of Disease Activity<br>• Assessment of how active a subject's PsA was on average during the last week<br>• Range, 0 (lowest level of disease activity) to 100 mm (highest level of disease activity)
**PtGA**	• Patient Global Assessment of Disease Activity<br>• Assesses how active a patient's PsA was on average during the last week<br>• Range 0-100 mm; higher scores indicate higher levels of disease activity / or a worse global health

C) Tolerability

**TEAEs**	• Treatment emergent adverse events<br>• Summarised as patient incidence and exposure-adjusted incidence rates (EAIR) per 100 patient years through Week 48 in patients receiving at least one dose of apremilast during FOREMOST (from randomization or transitioned from placebo) and through Week 16 in patients receiving at least one dose of blinded study drug (placebo or apremilast).

References

1 Schoels M, Aletaha D, Funovits J, Kavanaugh A, Baker D, Smolen JS. Application of the DAREA/DAPSA score for assessment of disease activity in psoriatic arthritis. *Ann Rheum Dis* 2010;69(8):1441-7.

2 Mease PJ, Gladman DD, Ogdie A, et al. Treatment-to-target with apremilast in psoriatic arthritis: the probability of achieving targets and comprehensive control of disease manifestations. *Arthritis Care Res (Hoboken)* 2020;72(6):814-21.

3 Gossec L, McGonagle D, Korotaeva T, et al. Minimal disease activity as a treatment target in psoriatic arthritis: a review of the literature. *J Rheumatol* 2018;45(1):6-13.

4 Coates LC, Fransen J, Helliwell PS. Defining minimal disease activity in psoriatic arthritis: a proposed objective target for treatment. *Ann Rheum Dis* 2010;69(1):48-53.

5 Coates LC, Strand V, Wilson H, et al. Measurement properties of the minimal disease activity criteria for psoriatic arthritis. *RMD Open* 2019;5(2):e001002.

6 Coates LC, Helliwell PS. Defining low disease activity states in psoriatic arthritis using novel composite disease instruments. *J Rheumatol* 2016;43(2):371-5.

7 Pincus T, Yazici Y, Bergman MJ. RAPID3, an index to assess and monitor patients with rheumatoid arthritis, without formal joint counts: similar results to DAS28 and CDAI in clinical trials and clinical care. *Rheum Dis Clin North Am* 2009;35(4):773-8, viii.

8 England BR, Tiong BK, Bergman MJ, et al. 2019 Update of the American College of Rheumatology Recommended Rheumatoid Arthritis Disease Activity Measures. *Arthritis Care Res (Hoboken)* 2019;71(12):1540-55.

Supplementary Table S2. Baseline demographics and disease characteristics of the overall FOREMOST population summarized by sex

	**Male (N=139)**	**Female (N=169)**	**Total (N=308)**
Age, mean (SD), years	50.1 (13.5)	51.6 (11.7)	50.9 (12.5)
Race, White, n (%)	130 (93.5)	161 (95.3)	291 (94.5)
PsA duration, months			
Mean (SD)	10.3 (10.5)	9.5 (9.9)	9.9 (10.2)
Median (Q1, Q3)	6.5 (3.7, 11.6)	6.0 (3.6, 11.7)	6.0 (3.7, 11.7)
SJC (0-66), mean (SD)	2.6 (0.7)	2.7 (0.7)	2.6 (0.7)
SJC category, n (%)			
2	73 (52.5)	77 (45.6)	150 (48.7)
3	51 (36.7)	66 (39.1)	117 (38.0)
4	15 (10.8)	26 (15.4)	41 (13.3)
TJC (0-68), mean (SD)	3.1 (0.8)	3.3 (0.8)	3.2 (0.8)
TJC category, n (%)			
2	36 (25.9)	28 (16.6)	64 (20.8)
3	58 (41.7)	57 (33.7)	115 (37.3)
4	45 (32.4)	84 (49.7)	129 (41.9)
Active joints,[a] mean (SD)	3.4 (1.0)	3.8 (0.8)	3.6 (0.9)
Active joints category, n (%)			
≤4	120 (86.3)	148 (87.6)	268 (87.0)
cDAPSA (0-154), mean (SD) [number of patients]	15.4 (4.3) [136]	16.8 (4.3) [167]	16.2 (4.4) [303]
PASDAS (0-10), mean (SD) [number of patients]	4.8 (1.0) [133]	5.1 (1.2) [166]	4.9 (1.1) [299]
PhGA (0-100 mm VAS), mean (SD)	43.5 (18.3) [135]	41.8 (19.2) [166]	42.6 (18.8) [301]
PtGA (0-100 mm VAS), mean (SD)	48.6 (20.4) [136]	53.4 (22.2) [167]	51.3 (21.5) [303]
PtGA >20, n (%)	121 (87.1)	151 (89.3)	272 (88.3)
LEI, mean (SD) [number of patients]	0.6 (1.2) [136]	1.1 (1.7) [167]	0.9 (1.5) [303]
LEI >1	23 (16.5)	45 (26.6)	68 (22.1)
Prior csDMARD, n (%)	93 (66.9)	111 (65.7)	204 (66.2)
Concomitant csDMARD, n (%)	60 (43.2)	63 (37.3)	123 (39.9)
Methotrexate	51 (36.7)	56 (33.1)	107 (34.7)
Sulfasalazine	9 (6.5)	7 (4.1)	16 (5.2)

Data reported as observed for N=308 randomised patients. [a]Active joints defined as swollen and/or tender.

cDAPSA, Clinical Disease Activity in Psoriatic Arthritis; csDMARD, conventional synthetic disease-modifying antirheumatic drugs; LEI, Leeds Enthesitis Index; PASDAS, Psoriatic Arthritis Disease Activity Score; PBO, placebo; PhGA, Physician's Global Assessment of Disease Activity; PsA, psoriatic arthritis; PtGA, Patient's Global Assessment of Disease Activity; Q1, quartile 1; Q3, quartile 3; RAPID 3; Routine Assessment of Patient Index Data 3; SD, standard deviation; SJC, swollen joint count; SPARCC, Spondyloarthritis Research Consortium of Canada Index for enthesitis; TJC, tender joint count; VAS, visual analogue scale.

Supplementary Table S3. Baseline demographics and disease characteristics of patients receiving ≥1 apremilast dose in FOREMOST

	PBO/APR (N=88)	APR (N=203)	Total (N=291)
Age, mean (SD), years	50.3 (13.0)	51.3 (12.3)	51.0 (12.5)
Sex, Women, n (%)	39 (44.3)	118 (58.1)	157 (54.0)
Race, White, n (%)	83 (94.3)	192 (94.6)	275 (94.5)
PsA duration, months			
Mean (SD)	10.6 (11.4)	9.8 (10.0)	10.0 (10.4)
Median (Q1, Q3)	6.0 (3.7, 13.5)	6.1 (3.7, 11.2)	6.1 (3.7, 11.8)
SJC (0-66), mean (SD)	2.5 (0.7)	2.7 (0.7)	2.6 (0.7)
SJC category, n (%)			
2	52 (59.1)	93 (45.8)	145 (49.8)
3	28 (31.8)	79 (38.9)	107 (36.8)
4	8 (9.1)	31 (15.3)	39 (13.4)
TJC (0-68), mean (SD)	3.2 (0.8)	3.2 (0.8)	3.2 (0.8)
TJC category, n (%)			
2	20 (22.7)	41 (20.2)	61 (21.0)
3	31 (35.2)	77 (37.9)	108 (37.1)
4	37 (42.0)	85 (41.9)	122 (41.9)
Active joints,[a] mean (SD)	3.5 (1.0)	3.6 (0.9)	3.6 (0.9)
Active joints category, n (%)			
≤4	78 (88.6)	176 (86.7)	254 (87.3)
Nail psoriasis, n (%)	59 (67.0)	143 (70.4)	202 (69.4)
PASS, n (%)	36 (40.9)	88 (43.3)	124 (42.6)
cDAPSA (0-154)			
Mean (SD) [number of patients]	15.6 (4.5) [88]	16.3 (4.3) [198]	16.1 (4.4) [286]
Remission, n (%)	0 (0)	0 (0)	0 (0)
Low disease activity, n (%)	28 (31.8)	45 (22.2)	73 (25.1)
Moderate disease activity, n (%)	60 (68.2)	153 (75.4)	213 (73.2)
High disease activity, n (%)	0 (0)	0 (0)	0 (0)
PASDAS (0-10), mean (SD) [number of patients]	4.8 (1.1) [87]	4.9 (1.1) [195]	4.9 (1.1) [282]
RAPID 3, mean (SD) [number of patients]	13.1 (5.4) [88]	13.7 (5.3) [197]	13.5 (5.4) [285]

	PBO/APR (N=88)	APR (N=203)	Total (N=291)
PhGA (0-100 mm VAS), mean (SD)	42.9 (19.5)	42.2 (18.6)	42.4 (18.9)
PtGA (0-100 mm VAS), mean (SD)	49.3 (20.7)	51.6 (22.0)	50.9 (21.6)
PtGA >20, n (%)	77 (87.5)	179 (88.2)	256 (88.0)
LEI, mean (SD) [number of patients]	2.5 (1.6) [30]	2.4 (1.5) [70]	2.4 (1.5) [100]
LEI >1	21 (23.9)	41 (20.2)	62 (21.3)
Prior csDMARD, n (%)	60 (68.2)	135 (66.5)	195 (67.0)
Concomitant csDMARD, n (%)	38 (43.2)	82 (40.4)	120 (41.2)
Methotrexate	31 (35.2)	73 (36.0)	104 (35.7)
Sulfasalazine	7 (8.0)	9 (4.4)	16 (5.5)

Data reported as observed for N=291 patients receiving at least one dose of APR. [a]Active joints defined as swollen and/or tender.

APR, apremilast; cDAPSA, Clinical Disease Activity in Psoriatic Arthritis; csDMARD, conventional synthetic disease-modifying antirheumatic drugs; LEI, Leeds Enthesitis Index; PASDAS, Psoriatic Arthritis Disease Activity Score; PBO, placebo; PASS, Patient Acceptable Symptom State; PhGA, Physician’s Global Assessment of Disease Activity; PsA, psoriatic arthritis; PtGA, Patient’s Global Assessment of Disease Activity; Q1, quartile 1; Q3, quartile 3; RAPID 3; Routine Assessment of Patient Index Data 3; SD, standard deviation; SJC, swollen joint count; SPARCC, Spondyloarthritis Research Consortium of Canada Index for enthesitis; TJC, tender joint count; VAS, visual analogue scale.

Supplementary Table S4. Baseline demographics and disease characteristics of csDMARD-experienced patients receiving ≥1 apremilast dose in FOREMOST

	PBO/APR (N=60)	APR (N=135)	Total (N=195)
Age, mean (SD), years	50.3 (13.1)	51.2 (12.7)	50.9 (12.8)
Sex, Women, n (%)	26 (43.3)	79 (58.5)	105 (53.8)
Race, White, n (%)	57 (95.0)	126 (93.3)	183 (93.8)
PsA duration, months			
Mean (SD)	12.9 (12.7)	11.6 (10.4)	12.0 (11.1)
Median (Q1, Q3)	7.0 (4.3, 15.5)	8.0 (4.7, 14.7)	7.9 (4.6, 15.0)
SJC (0-66), mean (SD)	2.4 (0.6)	2.7 (0.7)	2.6 (0.7)
SJC category, n (%)			
2	37 (61.7)	62 (45.9)	99 (50.8)
3	20 (33.3)	55 (40.7)	75 (38.5)
4	3 (5.0)	18 (13.3)	21 (10.8)
TJC (0-68), mean (SD)	3.1 (0.8)	3.2 (0.8)	3.2 (0.8)
TJC category, n (%)			
2	14 (23.3)	28 (20.7)	42 (21.5)
3	25 (41.7)	51 (37.8)	76 (39.0)
4	21 (35.0)	56 (41.5)	77 (39.5)
Active joints,[a] mean (SD)	3.3 (1.0)	3.6 (0.8)	3.5 (0.9)
≤4	55 (91.7)	120 (88.9)	175 (89.7)
cDAPSA ModDA, n (%)	42 (70.0)	99 (73.3)	141 (72.3)
RAPID 3, mean (SD)	13.3 (5.3)	13.5 (5.1)	13.4 (5.2)
PhGA (0-100 mm VAS), mean (SD)	43.6 (18.2)	44.5 (17.5)	44.2 (17.6)
PtGA (0-100 mm VAS), mean (SD)	52.2 (20.1)	51.1 (20.7)	51.4 (20.5)
PtGA >20, n (%)	55 (91.7)	120 (88.9)	175 (89.7)
LEI (0-6), mean (SD) [number of patients]	0.8 (1.5) [60]	0.7 (1.3) [132]	0.7 (1.4) [192]
LEI >1	13 (21.7)	23 (17.0)	36 (18.5)
Concomitant csDMARD, n (%)	38 (63.3)	81 (60.0)	119 (61.0)
Methotrexate	31 (51.7)	72 (53.3)	103 (52.8)
Sulfasalazine	7 (11.7)	9 (6.7)	16 (8.2)

Data reported as observed for N=195 patients with prior csDMARD use receiving at least one dose of APR. [a]Active joints defined as swollen and/or tender.

APR, apremilast; cDAPSA, Clinical Disease Activity in Psoriatic Arthritis; csDMARDs, conventional synthetic disease-modifying antirheumatic drugs; LEI, Leeds Enthesitis Index; PASDAS, Psoriatic Arthritis Disease Activity Score; PBO, placebo; PhGA, Physician's Global Assessment of Disease Activity; PsA, psoriatic arthritis; PtGA, Patient's Global Assessment of Disease Activity; Q1, quartile 1; Q3, quartile 3; RAPID 3; Routine Assessment of Patient Index Data 3; SD, standard deviation; SJC, swollen joint count; SPARCC, Spondyloarthritis Research Consortium of Canada Index for enthesitis; TJC, tender joint count; VAS, visual analogue scale.

Supplementary Table S5. Summary of TEAEs in patients receiving ≥1 apremilast dose in FOREMOST

	TEAEs through Week 48[a]		TEAEs through Week 16[b]			
	N=291 Patients Receiving at Least One Dose of APR Pt-years=192.5		PBO N=104 Pt-years=30.0		APR N=204 Pt-years=57.5	
	n (%)	EAIR/100 Pt-yrs	n (%)	EAIR/100 Pt-yrs	n (%)	EAIR/100 Pt-yrs
Any TEAE	185 (63.6)	184.1	44 (42.3)	207.1	102 (50.0)	277.5
Subject incidence ≥5%[c]						
Diarrhoea	69 (23.7)	44.5	7 (6.7)	24.8	45 (22.1)	93.1
Nausea	28 (9.6)	15.5	3 (2.9)	10.2	20 (9.8)	37.2
Headache	21 (7.2)	11.5	2 (1.9)	6.7	13 (6.4)	23.7
COVID-19	19 (6.5)	10.2	3 (2.9)	10.2	5 (2.5)	8.7
Any severe TEAE	14 (4.8)	7.4	3 (2.9)	10.1	6 (2.9)	10.6
Any serious TEAE	15 (5.2)	7.9	4 (3.8)	13.5	7 (3.4)	12.2
TEAEs leading to drug interruption	24 (8.2)	13.1	2 (1.9)	6.7	10 (4.9)	17.8
TEAEs leading to drug withdrawal	28 (9.6)	14.7	5 (4.8)	16.7	19 (9.3)	33.7
TEAEs leading to death[d]	2 (0.7)[d]	1.0	0 (0)	0	1 (0.5)[d]	1.7

APR, apremilast; EAIR, exposure-adjusted incidence rate, defined as 100 times the number (n) of patients reporting the event divided by total patient-years (up to the first event start date for subjects reporting the event); PBO, placebo; TEAE, treatment-emergent adverse event.
[a]Includes patients randomised to APR and patients who transitioned from PBO to APR at Week 16 or 24; summarises TEAEs with a start date on or after the first dose of APR and no later than 28 days after the last dose of APR or the date of death, whichever comes first.

[b]Summarises TEAEs with a start date on or after the first dose of study drug (APR or PBO) and no later than Week 16 or the date of death, whichever comes first, or 28 days after the last dose of APR for patients who withdrew before Week 16.
[c]TEAEs selected based on subject incidence in N=291 patients who received at least one dose of APR.
[d]Two deaths were reported in the APR group before the open-label extension phase of the study: a sudden cardiac death on Day 82, relative to Day 1 of APR and an anoxic brain injury (following a planned routine abdominal herniorrhaphy) on Day 240, with a last dose of APR on Day 151. Neither death was related to study drug as assessed by the investigator.

Supplementary Figure S1. FOREMOST Study design

Alt text: Study schema showing a 24-week randomised, double-blind, placebo-controlled phase followed by open-label apremilast through week 48, early escape at week 16, and standard of care permitted throughout the study.

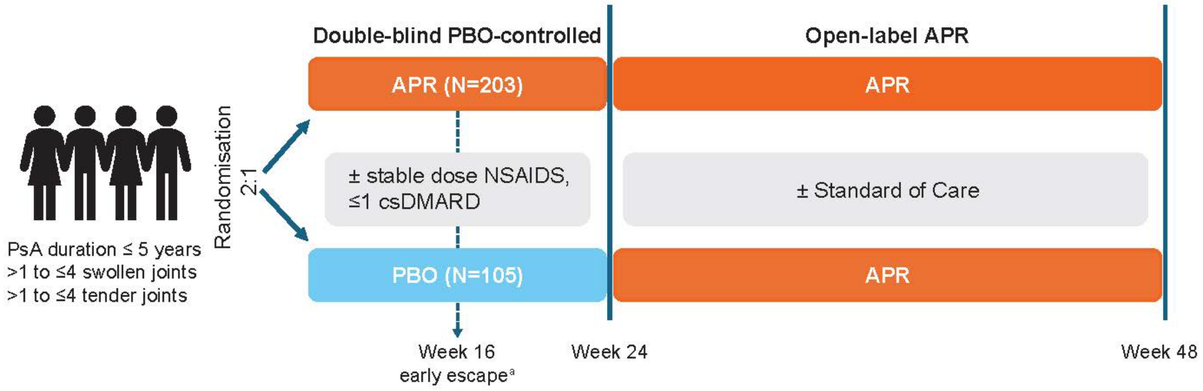

[a]Patients who had no improvement in SJC at Week 16 were eligible for early escape at Week 16 and could transition to APR; patients initially randomised to APR continued to receive APR.
APR, apremilast 30 mg twice daily; csDMARD, conventional synthetic disease-modifying antirheumatic drug; NSAID, non-steroidal anti-inflammatory drug; PBO, placebo; SJC, swollen joint count.

Supplementary Figure S2. Covariates included in multivariable regression of progression from oligoarticular to polyarticular PsA at Week 16

Alt text: Table listing treatment (apremilast versus placebo), sex (male versus female), age (younger than 50 versus 50 or older); baseline BMI (less than 30 versus greater than or equal to 30 kg per meter squared); prior csDMARDs (yes versus no); concomitant cDMARDs (yes versus no); baseline cDAPSA (low versus moderate disease activity); baseline patient pain VAS (less than or equal to 53 versus greater than 53); baseline HAQ-DI (less than or equal to 0.5 versus greater than 0.5); Patient Acceptable Symptom State at baseline (yes versus no); baseline PASDAS (low disease activity/remission versus moderate/high disease activity); baseline patient global assessment VAS (less than or equal to 50,versus greater than 50), baseline Leeds enthesitis index (0 versus greater than 0); baseline SPARCC (0 versus greater than 0); baseline Leeds dactylitis Index (0 versus greater than 0); baseline nail psoriasis (yes versus no).

**Treatment**	APR, PBO
**Sex**	male, female
**Age**	<50 years, ≥50 years
**BMI**	<30 kg/m^2, ≥30 kg/m^2
**Prior csMARDS**	yes, no (csDMARD naïve)
**csDMARDs on study**	yes, no
**Baseline DAPSA**	>4 to ≤13 (low disease activity), >13 to ≤27 (moderate disease activity)
**Patient pain VAS**	≤median (=53), >median
**Baseline HAQ-DI**	≤0.5 (remission disease control), >0.5
**Baseline PASS**	yes, no
**Baseline PASDAS**	<5.4 (low disease activity), ≥5.4
**Baseline PtGA VAS**	≤50, >50
**Baseline LEI**	0 (no enthesitis), >0
**Baseline SPARCC**	0 (no enthesitis), >0
**Baseline LDI**	0 (no dactylitis), >0
**Nail psoriasis at baseline**	yes, no

Progression from oligoarticular to polyarticular PsA defined as moving from ≤4 active (swollen and/or tender) joints at baseline to >4 active joints post-baseline.

APR, apremilast; BMI, body mass index; csDMARD; conventional systemic antirheumatic drug (methotrexate or sulfasalazine); cDAPSA, clinical Disease Activity in Psoriatic Arthritis; HAQ-DI, Health Assessment Questionnaire Disability Index; LDI, Leeds Dactylitis Index; LEI, Leeds Enthesitis Index; PASDAS, Psoriatic Arthritis Disease Activity Score; PASS, Patient Acceptable Symptom State; PBO, placebo; PtGA, patient global assessment of disease activity; SPARCC, Spondylarthritis Research Consortium of Canada; VAS, visual analog scale.

Supplementary Figure S3. Swollen, Tender and Active Joints in patients receiving ≥1 apremilast dose in FOREMOST

Alt text: Line graphs labelled A to C showing mean swollen, tender and active joints through week 48, summarised for patients randomised to placebo and transitioning to apremilast versus patients receiving apremilast from randomisation.

A) Mean Swollen Joint Count

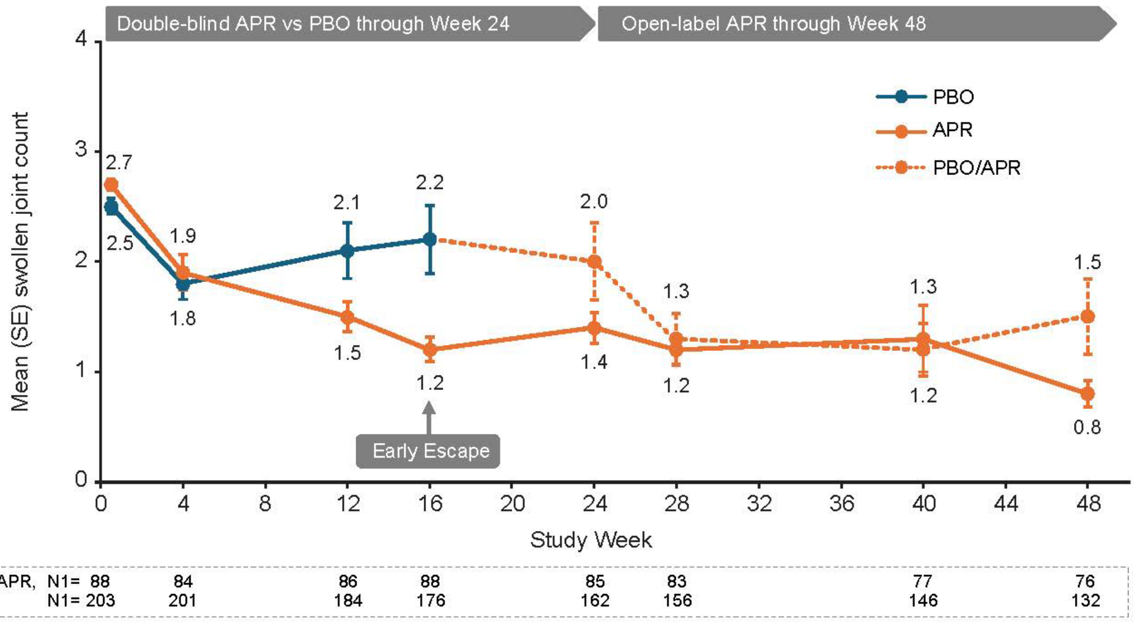

B) Mean Tender Joint Count

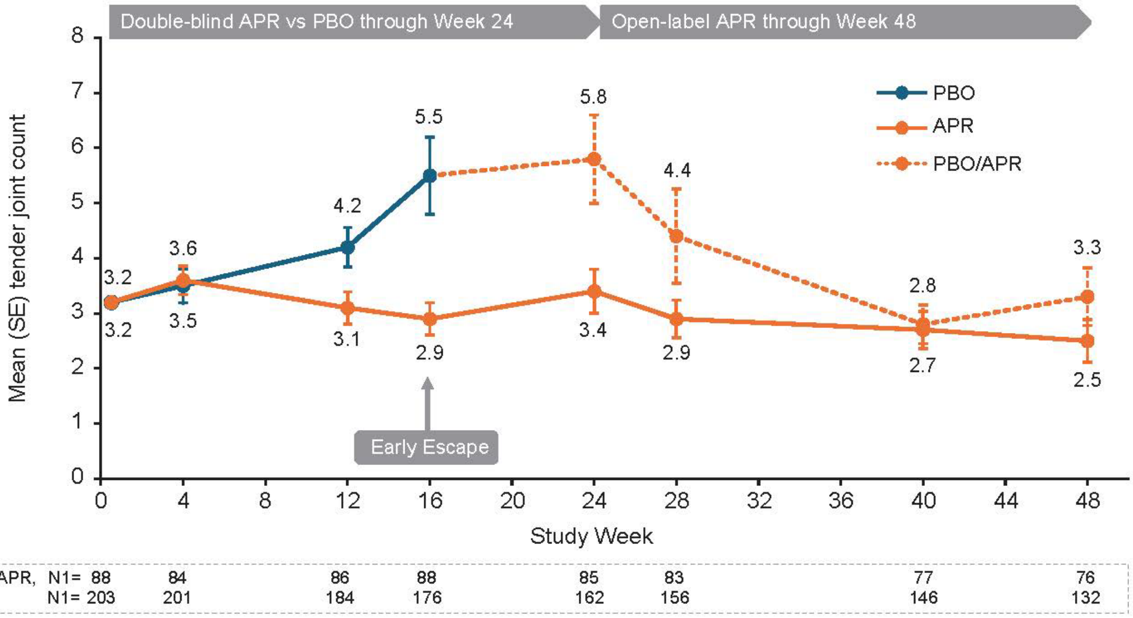

C) Mean Active Joint Count

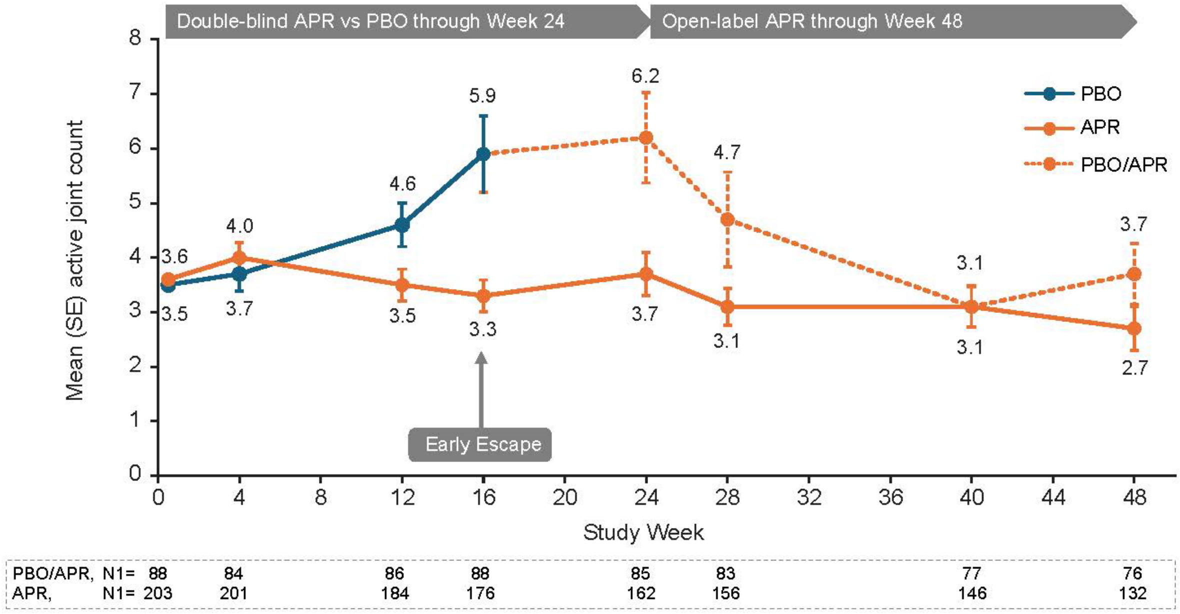

Data reported as observed for N=291 patients receiving at least one dose of APR. "APR" denotes data through Week 48 for patients receiving APR from randomisation. For patients transitioning from PBO to APR, "PBO" denotes data from baseline to Week 16 and "PBO/APR" denotes data after Week 16. APR, apremilast; N1, number of patients with non-missing data at each timepoint; PBO, placebo; SE, standard error.

Supplementary Figure S4. Discontinuations among patients receiving ≥1 apremilast dose in FOREMOST

Alt text: Flow chart for 291 patients receiving at least one apremilast dose, with 203 randomised to apremilast, 24 randomised to placebo and transitioning to apremilast at week 16, 64 randomised to placebo and transitioning to apremilast at week 24, 36 discontinuing by week 24, 5 completing week 24 and discontinuing before entering the study extension, 249 entering the extension and 39 discontinuing during the extension.

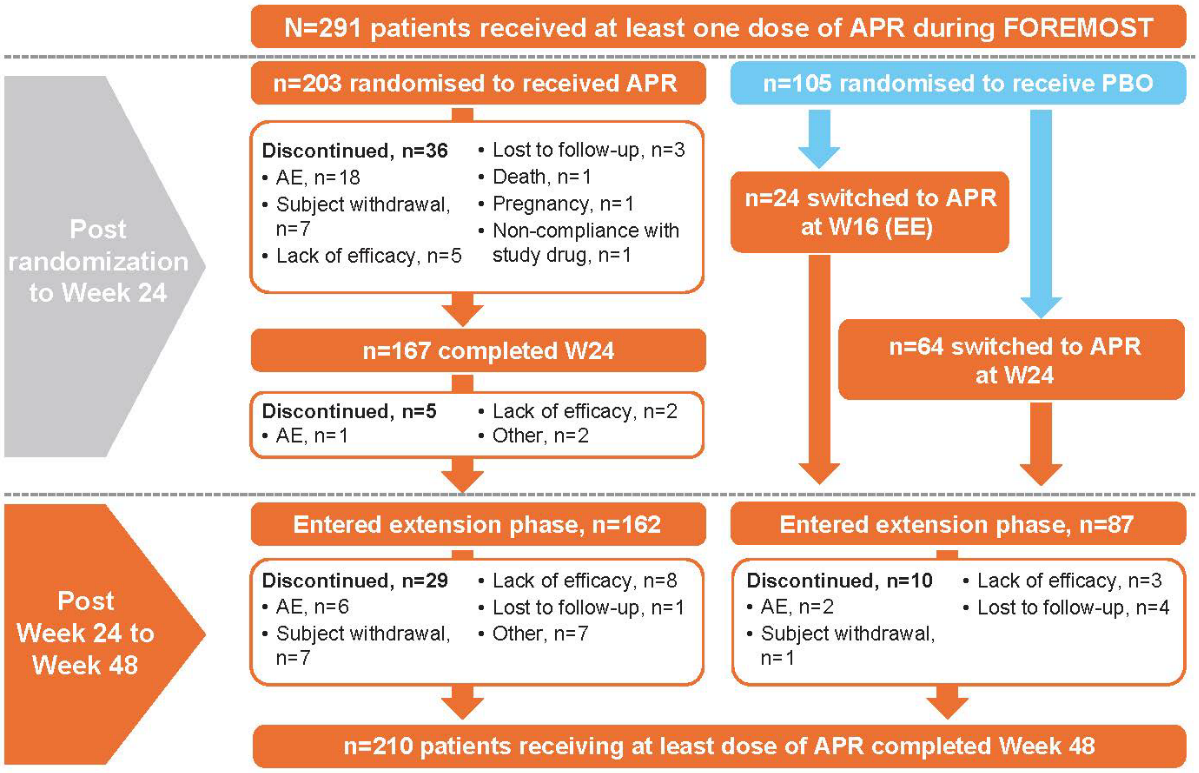

Data reported as observed for N=291 patients receiving at least one dose of APR. One patient who transitioned from PBO to APR at Week 16 completed Week 24 and did not enter the extension phase. Discontinuations among patients receiving PBO through Week 24 and not entering the extension phase have been reported previously.

AE, adverse event; APR, apremilast; EE, early escape; PBO, placebo; SE, standard error.

Supplementary Figure S5. Improvements in enthesitis in patients receiving ≥1 apremilast dose in FOREMOST

Alt text: Line graphs labelled A to C showing the percentage of patients moving from a Leeds enthesitis index above zero to a value of zero, and mean changes in Leeds enthesitis index and SPARCC through week 48, summarised for patients randomised to placebo and transitioning to apremilast versus patients receiving apremilast from randomisation.

A) Percentage of patients moving from LEI>0 to LEI=0

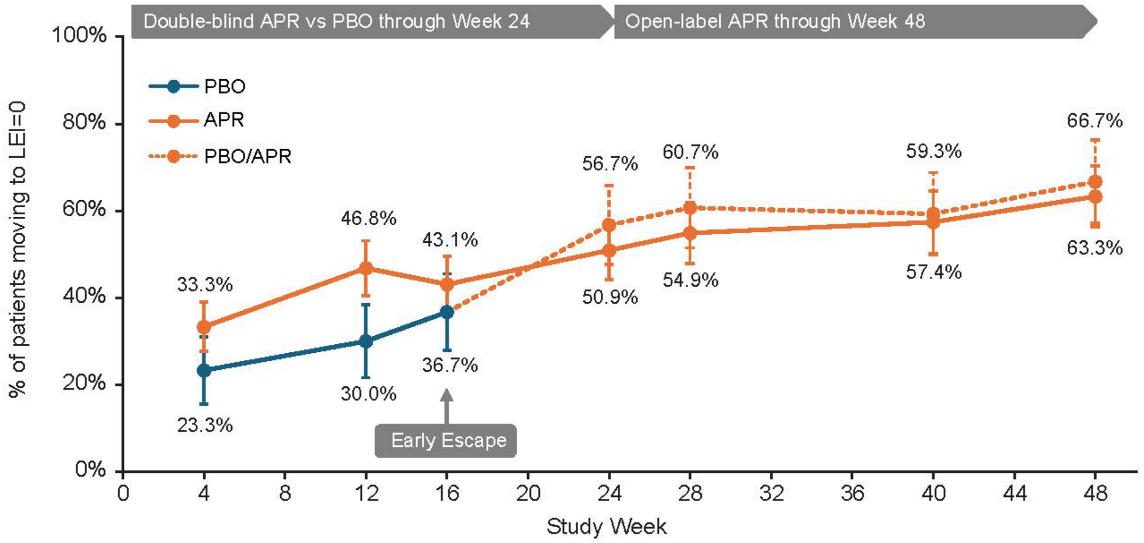

	4	12	16	24	28	40	48
PBO/APR, n/N1=	7/30	9/30	11/30	17/30	17/28	16/27	16/24
APR, n/N1=	23/69	29/62	25/58	28/55	28/51	27/47	31/49

B) Mean change from baseline LEI in patients with baseline LEI>0

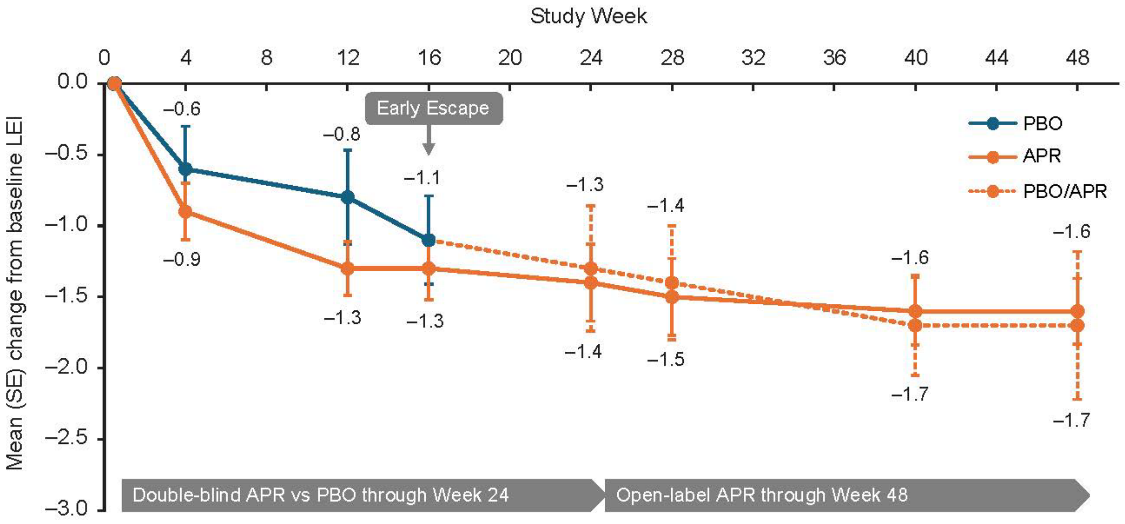

	4	12	16	24	28	40	48
PBO/APR, N1=	30	30	30	30	28	27	24
APR, N1=	69	62	58	55	51	47	49

C) Percentage of patients moving from SPARCC>0 to SPARCC=0

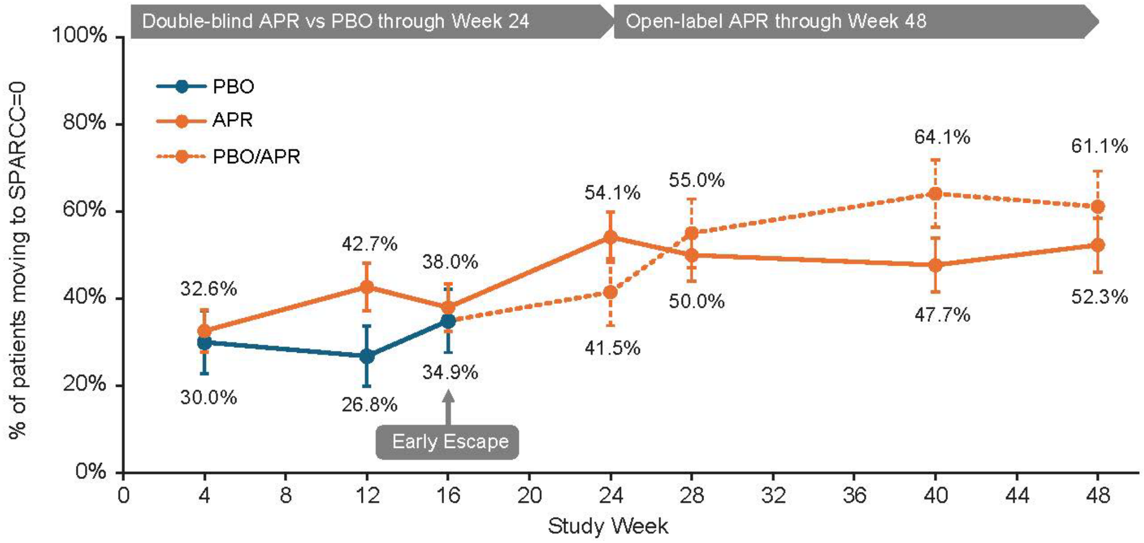

PBO/APR,	n/N1=	12/40	11/41	15/43	17/41	22/40	25/39	22/36
APR,	n/N1=	30/92	35/82	30/79	40/74	35/70	31/65	34/65

A) and B) report data as observed for N=100 patients with baseline LEI >0 receiving at least one dose of APR. C) reports data as observed for N=137 patients with baseline SPARCC >0 receiving at least one dose of APR.

“APR” denotes data through Week 48 for patients receiving APR from randomisation. For patients transitioning from PBO to APR, “PBO” denotes data from baseline to Week 16 and “PBO/APR” denotes data after Week 16.

APR, apremilast; LEI, Leeds Enthesitis Index; n=number of patients achieving the outcome of interest at each timepoint; N1, number of patients with non-missing data at each timepoint; PBO, placebo; SE, standard error; SPARCC, Spondylarthritis Research Consortium of Canada.

Supplementary Figure S6. Improvements in Physician and Patient Global Assessment in patients receiving ≥1 apremilast dose in FOREMOST

Alt text: Line graphs labelled A and B showing mean changes in Physician and Patient Global Assessments through week 48, summarised for patients randomised to placebo and transitioning to apremilast versus patients receiving apremilast from randomisation.

A) Mean Change in Physician Global Assessment

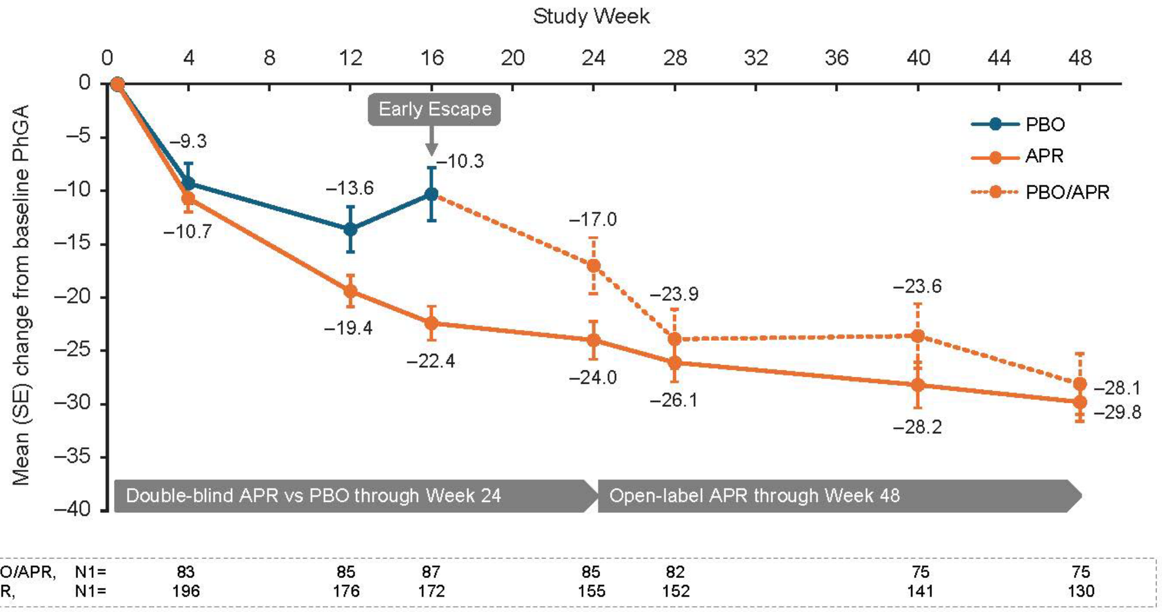

B) Mean change In Patient Global Assessment

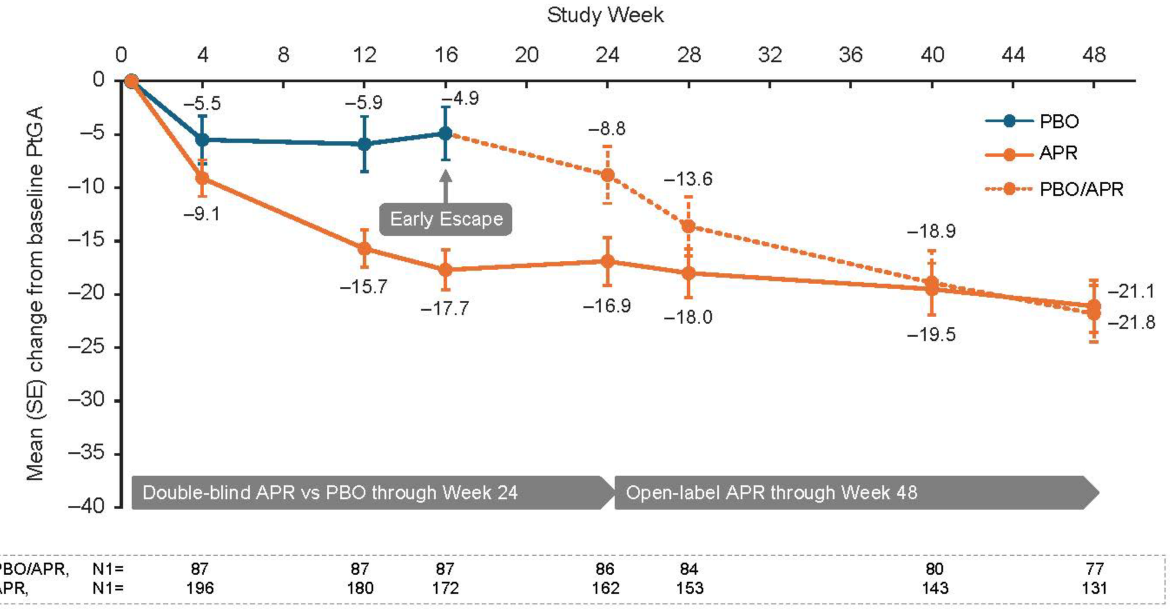

Data reported as observed for N=291 patients receiving at least one dose of APR.

“APR” denotes data through Week 48 for patients receiving APR from randomisation. For patients transitioning from PBO to APR, “PBO” denotes data from baseline to Week 16 and “PBO/APR” denotes data after Week 16.

APR, apremilast; N1, number of patients with non-missing data at each timepoint; PBO, placebo; PtGA, Patient Global Assessment; SE, standard error; PhGA, Physician Global Assessment.

Figures for csDMARD-experienced patients

Supplementary Figure S7. Disease progression in csDMARD experienced patients receiving ≥1 apremilast dose in FOREMOST

Alt text: Line graphs labelled A to C showing the percentage patients with progression and mean swollen and tender joint counts through week 48, summarised for patients randomised to placebo and transitioning to apremilast versus patients receiving apremilast from randomisation.

A) Progression from oligoarticular PsA to polyarticular PsA

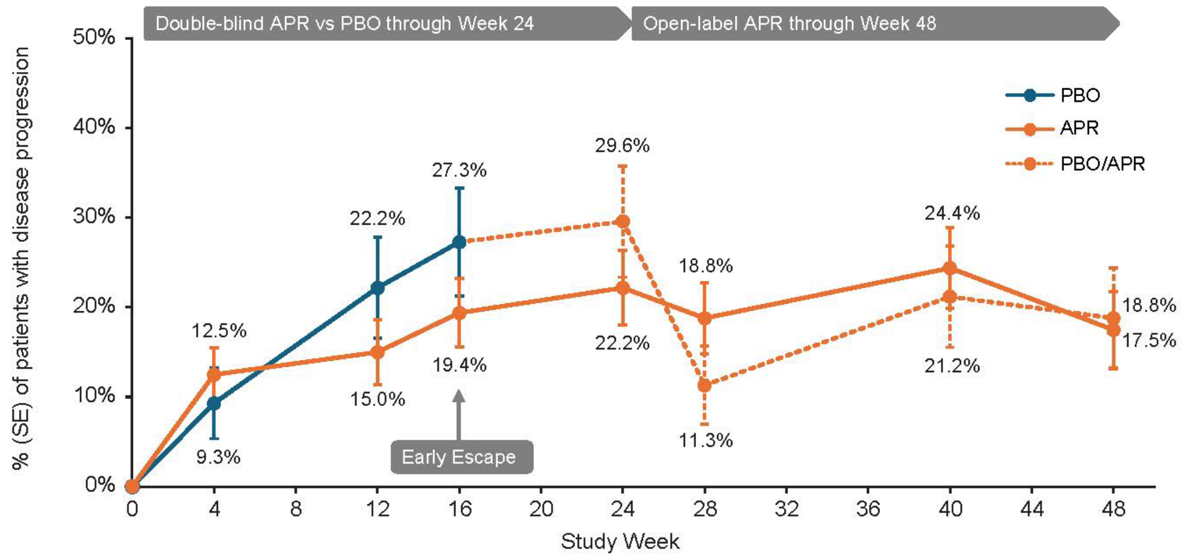

		4	12	16	24	28	40	48
PBO/APR,	n/N1=	5/54	12/54	15/55	16/54	6/53	11/52	9/48
APR,	n/N1=	15/120	17/113	21/108	22/99	18/96	22/90	14/80

B) Mean Swollen Joint Count

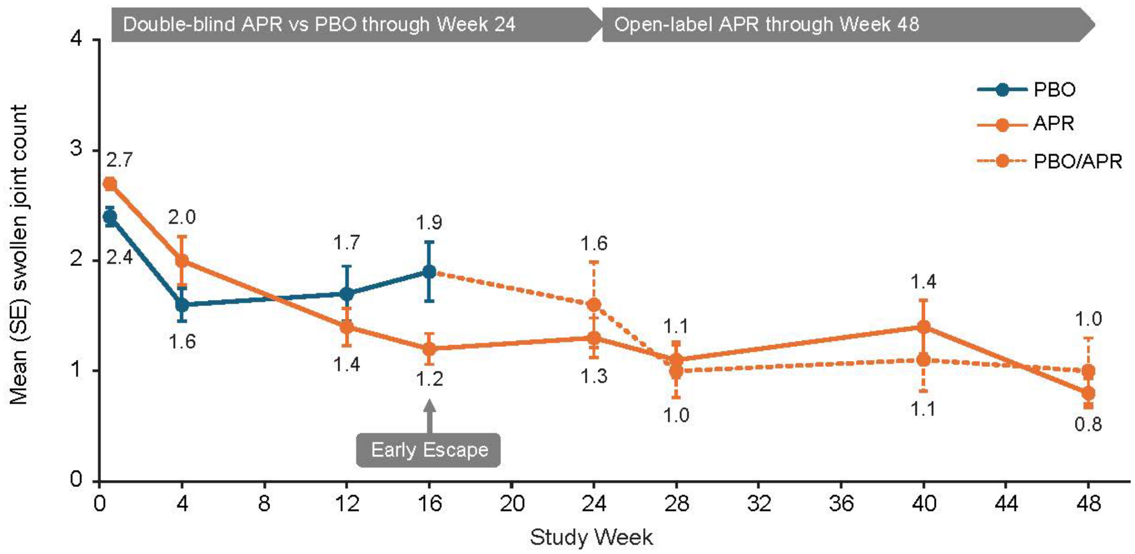

	0	4	12	16	24	28	40	48
PBO/APR, N1=	60	59	59	60	59	58	56	52
APR, N1=	135	135	128	122	112	109	103	93

C) Mean Tender Joint Count

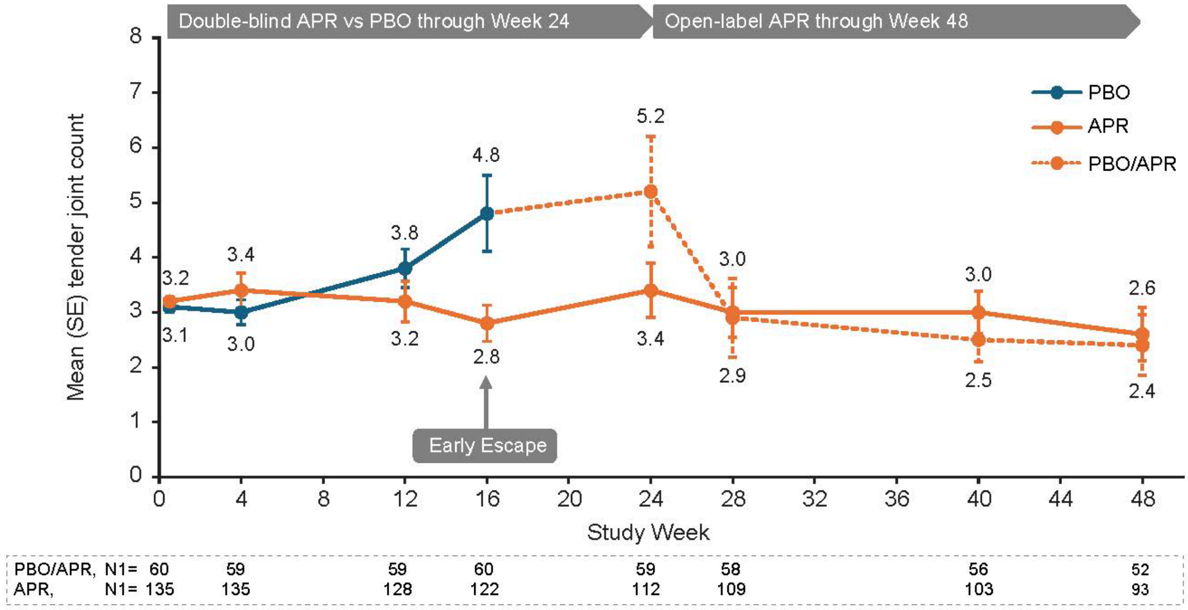

Data reported as observed for A) N=175 csDMARD experienced patients with ≤4 active (swollen and/or tender) joints at baseline receiving at least one dose of APR; B), C) N=195 csDMARD experienced patients receiving at least one dose of APR. Progression from oligoarticular to polyarticular PsA defined as moving from ≤4 active joints at baseline to >4 active joints post-baseline

"APR" denotes data through Week 48 for patients receiving APR from randomisation. For patients transitioning from PBO to APR, "PBO" denotes data from baseline to Week 16 and "PBO/APR" denotes data after Week 16.

APR, apremilast; csDMARD, conventional synthetic disease-modifying antirheumatic drug; n, number of patients with disease progression at each time point; N1, number of patients with non-missing data at each timepoint; PBO, placebo; SE, standard error.

Supplementary Figure S8. Achievement of disease activity goals in csDMARD experienced patients receiving ≥1 apremilast dose in FOREMOST

Alt text: Bar charts labelled A to F showing the percentage of patients achieving MDA, MDA joints, cDAPSA remission or low disease activity, PASDAS moderate or good response, very low disease activity, and RAPID3 minimal clinically important difference at weeks 16 and 48, summarised for patients randomised to placebo and transitioning to apremilast versus patients receiving apremilast from randomisation.

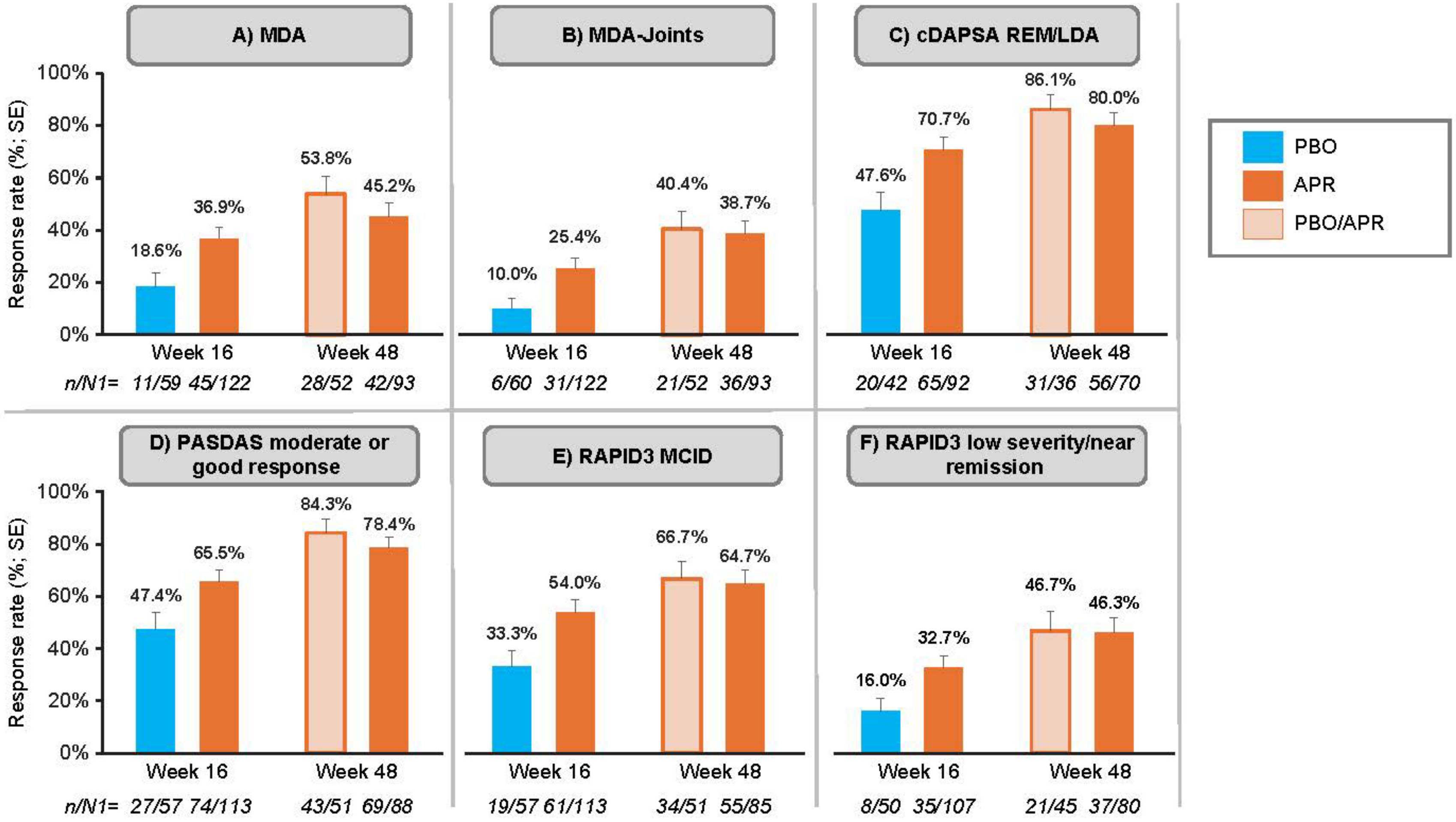

A), B), and D) report data as observed for N=195 csDMARD experienced patients receiving at least one dose of APR. C) reports data as observed for N=141 csDMARD experienced patients with baseline cDAPSA High or Moderate Disease Activity receiving at least one dose of APR. E) reports data as observed for N=184 csDMARD experienced patients with baseline RAPID3 score ≥3.8 receiving at least one dose of APR. F) reports data as observed for N=170 csDMARD experienced patients with baseline RAPID3 score >6 (high/moderate severity) receiving at least one dose of APR.
APR denotes Week 16 and Week 48 data in patients receiving APR from randomisation. PBO denotes Week 16 data in patients randomised to receive PBO through Week 24 (early escape at Week 16). PBO/APR denotes Week 48 data in patients randomised to PBO who transitioned to APR at Week 16 or 24.

APR, apremilast; cDAPSA, Clinical Disease activity in Psoriatic Arthritis; LDA, low disease activity; MCID, Minimally Important Clinical Difference; MDA, Minimal Disease Activity; MDA-Joints, Modified MDA; n, number of patients achieving disease activity goal; N1=number of patients with non-missing data; PASDAS, Psoriatic Arthritis Disease Activity Score; PBO, placebo; REM, remission; SE, standard error; VLDA, very low disease activity.
